# Supplementary material for: Helix-bundle and C-terminal GPCR domains differentially influence GRK-specific functions and β-arrestin-mediated regulation
Source: Nat Commun. 2025 Jul 1;16:5430. doi: 10.1038/s41467-025-61281-4 (PMC12214593; doi:10.1038/s41467-025-61281-4)
Supplement: Supplementary file 1 — Supplementary Information [file 41467_2025_61281_MOESM1_ESM.pdf]

Helix-bundle and C-terminal GPCR domains differentially influence GRK-specific functions and β-arrestin-mediated regulation

Edda S. F. Matthees*, Raphael S. Haider*, Laura Klement, Mona Reichel, Nina K. Blum, Verena Weitzel, Thimea Trüpschuch, Carla Ziegler, Julia Drube, Stefan Schulz and Carsten Hoffmann

* contributed equally
Corresponding author: Prof. Dr. Carsten Hoffmann (carsten.hoffmann@med.uni-jena.de)

Supplementary material
Including Suppl. Fig. 1-12 and Suppl. Tab. 1-6, as well as the respective legends

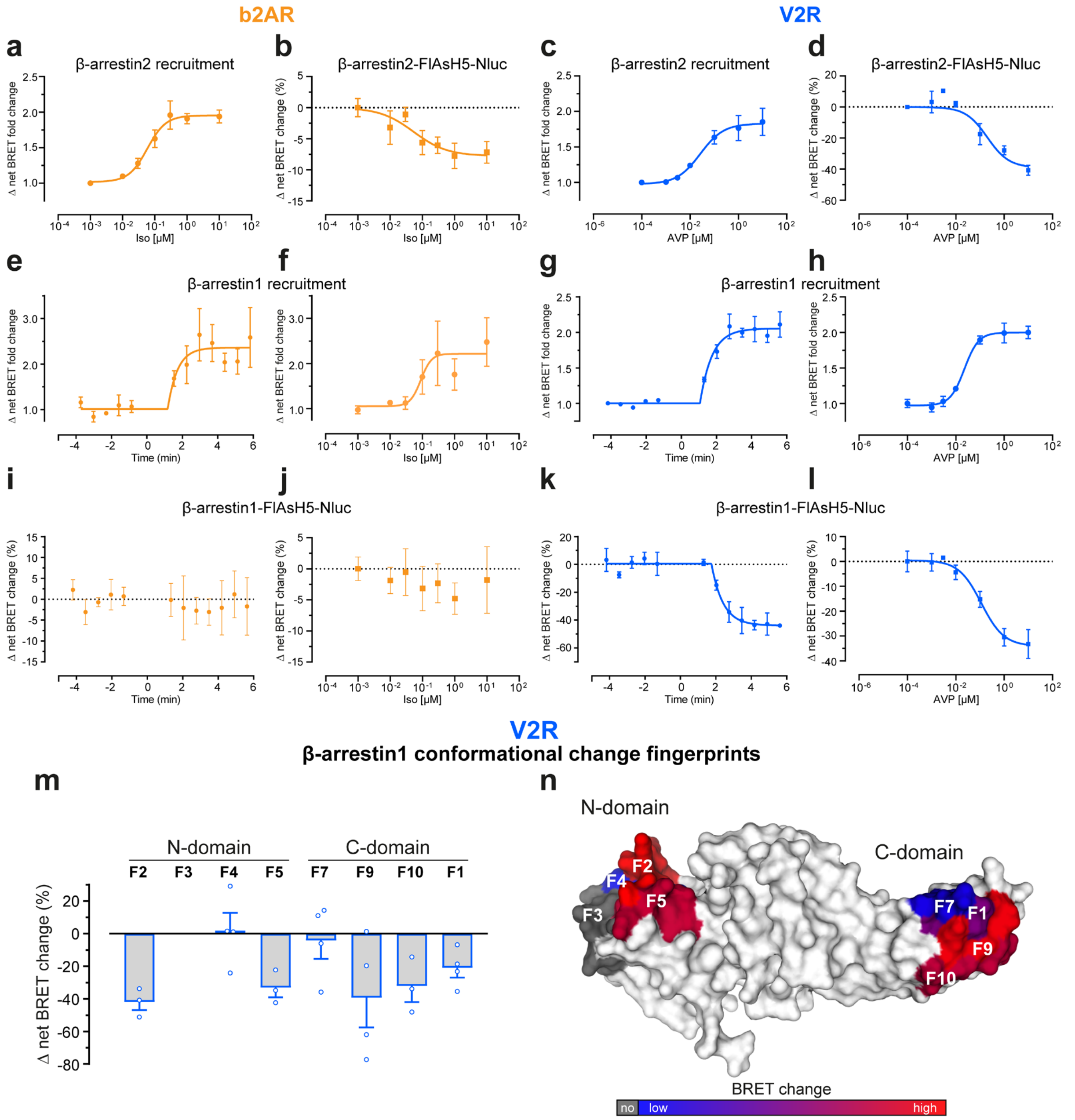

Supplementary Figure 1: b2AR and V2R differentially interact with β-arrestin1 and 2. a-d, β-arrestin2 recruitment to b2AR (**a**) and V2R (**c**, data initially published in Drube *et al.*[26]), as well as conformational change of β-arrestin2-FlAsH (F) 5-Nluc in Control cells, co-transfected with untagged b2AR (**b**) or V2R (**d**), as concentration-response curves. Concentration-dependent values correspond to data shown in **Fig.1 b, c, e, f**, averaging timepoints between 2-4 min after stimulation with the indicated agonist concentration. **e-l,** β-arrestin1 recruitment to b2AR (**e, f**) and V2R (**g, h**, data initially published in Drube *et al.*[26]), and β-arrestin1-F5-Nluc conformational changes (**i-l**) were measured in Control cells. BRET changes are shown over time (**e, g, i, k**) following stimulation with 10 µM Iso (e, i) or 3 µM AVP (g, k) or in a concentration-dependent manner (**f, h, j, l**). Data were analyzed analogously to measurements with β-arrestin2 (**Fig.1a-f** and **a-d**) and are shown as Δ net BRET fold change (**a, c, e-h**) or Δ net BRET change (%) (**b, d, i-l**) ± SEM of at least $n=3$ independent experiments. **m, n,** Fingerprint of β-arrestin1 conformational change sensors measured in Control cells in presence of untagged V2R. The Δ net BRET changes at 3 µM AVP are shown as bar graphs (**m**). Sensor conditions, which did not fulfill the set pharmacological parameters (Hill slope and EC_{50} analysis as described in methods and **Suppl. Fig. 2**) were classified as non-responding positions and assigned zero. These data were projected onto the surface of the inactive β-arrestin1 crystal structure (PDB: 2WTR) by coloring the respective loop (-fragments) of the labeled FlAsH positions (**n**) ranging from blue to red. The Δ net BRET change was normalized to the maximum sensor signal (red). Non-responding conditions are shown in gray.

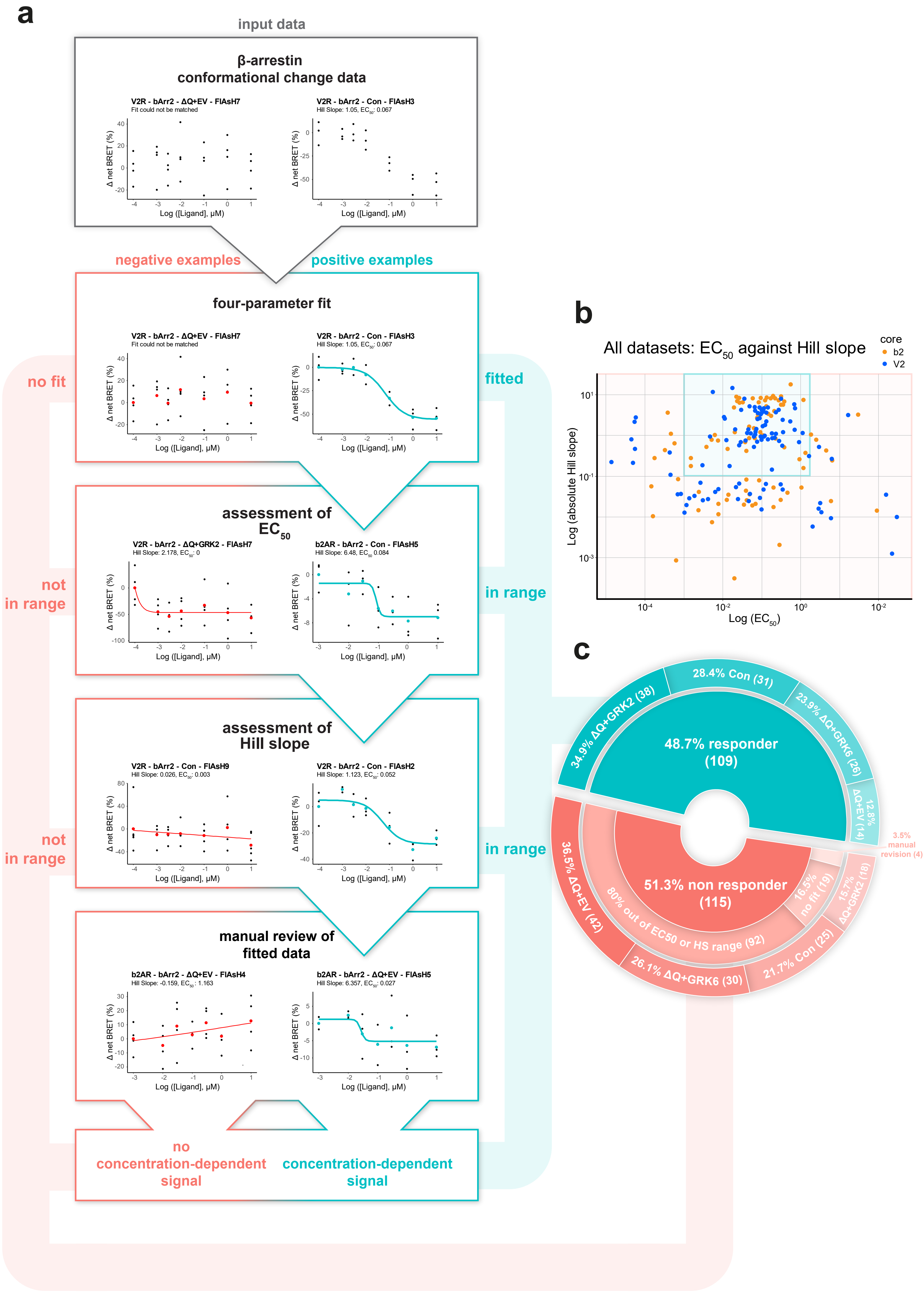
a
input data
β-arrestin conformational change data
V2R - bArr2 - ΔQ+EV - FlAsH7
Fit could not be matched
V2R - bArr2 - Con - FlAsH3
Hill Slope: 1.05, EC50: 0.067
Δ net BRET (%)
Log ([Ligand], μM)
negative examples
positive examples
four-parameter fit
no fit
fitted
assessment of EC50
V2R - bArr2 - ΔQ+GRK2 - FlAsH7
Hill Slope: 2.178, EC50: 0
b2AR - bArr2 - Con - FlAsH5
Hill Slope: 6.48, EC50: 0.084
not in range
in range
assessment of Hill slope
V2R - bArr2 - Con - FlAsH9
Hill Slope: 0.026, EC50: 0.003
V2R - bArr2 - Con - FlAsH2
Hill Slope: 1.123, EC50: 0.052
not in range
in range
manual review of fitted data
b2AR - bArr2 - ΔQ+EV - FlAsH4
Hill Slope: -0.159, EC50: 1.163
b2AR - bArr2 - ΔQ+EV - FlAsH5
Hill Slope: 6.357, EC50: 0.027
no concentration-dependent signal
concentration-dependent signal
b
All datasets: EC50 against Hill slope
core
b2
V2
Log (absolute Hill slope)
Log (EC50)
c
48.7% responder (109)
51.3% non responder (115)
28.4% Con (31)
23.9% ΔQ+GRK6 (26)
12.8% ΔQ+EV (14)
34.9% ΔQ+GRK2 (38)
3.5% manual revision (4)
16.5% no fit (19)
80% out of EC50 or HS range (92)
36.5% ΔQ+EV (42)
26.1% ΔQ+GRK6 (30)
21.7% Con (25)
15.7% ΔQ+GRK2 (18)

Supplementary Figure 2: Pharmacological analysis route for the interpretation of biosensor signals. a, Flowchart detailing individual steps of data analysis to identify "responding" and "non-responding" β-arrestin conformational change signals (featuring different example datasets for results that were eliminated in different steps of the analysis pipeline). In brief, concentration-dependent data of at least n=3 independent experiments served as the input and were fitted using a non-linear four parameter model. Subsequently, fitting parameters (EC_{50} and Hill slope) were assessed and characterized according to set constraints (Hill slope absolute value > 0.1, EC_{50} between 10-3 and 100.3 µM). Finally, all datasets were subjected to manual review. Data for which a four-parameter fit was not applicable and/or did not adhere to set constraints were classified as "non-responding" and hence interpreted as 0% conformational change throughout the study. **b,** Scatterplot showing EC_{50} and absolute Hill slope values of all measured conditions. Set constraints used to identify "responding" β-arrestin conformational change signals are indicated with a green box. **c,** Donut plot that shows the makeup and characterization of all β-arrestin conformational change data included in this study, detailing the exact reasons for "non-responder" identification, as well as the individual fractions of cellular GRK-specific conditions.

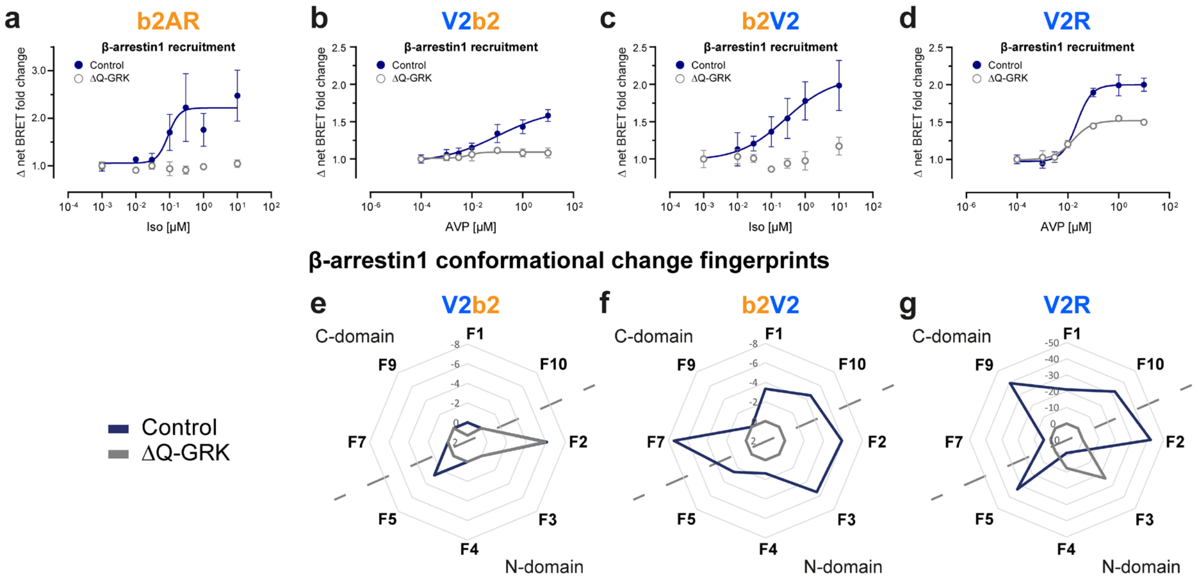

Supplementary Figure 3: Different combinations of GPCR helix bundles and C-termini orchestrate distinct, GRK-dependent β-arrestin1 interactions and conformational changes. a-d, β-arrestin1 recruitment to the b2AR (**a**), V2b2 (**b**), b2V2 (**c**) and V2R (**d**) in ΔQ-GRK or Control cells stimulated with Iso (**a, c**) or AVP (**b, d**) as indicated. Data for b2AR, b2V2 and V2R were initially published in Drube *et al.*[26] and are shown again to allow a direct comparison. Data are shown as Δ net BRET fold change over baseline ± SEM of *n*=3 independent experiments. **e-g,** Complete β-arrestin1 conformational change fingerprints are shown for each receptor in Control and ΔQ-GRK cells, stimulated with 10 µM Iso (**f**) or 3 µM AVP (**e, g**), as Δ net BRET change (%) as radar plots, analogously to the data for β-arrestin2 (**Fig. 2s-v**). Sensor conditions, which did not fulfill the pharmacological parameters (Hill slope and EC_{50} analysis as described in methods and **Suppl. Fig. 2**) were classified as non-responding conditions and assigned zero.

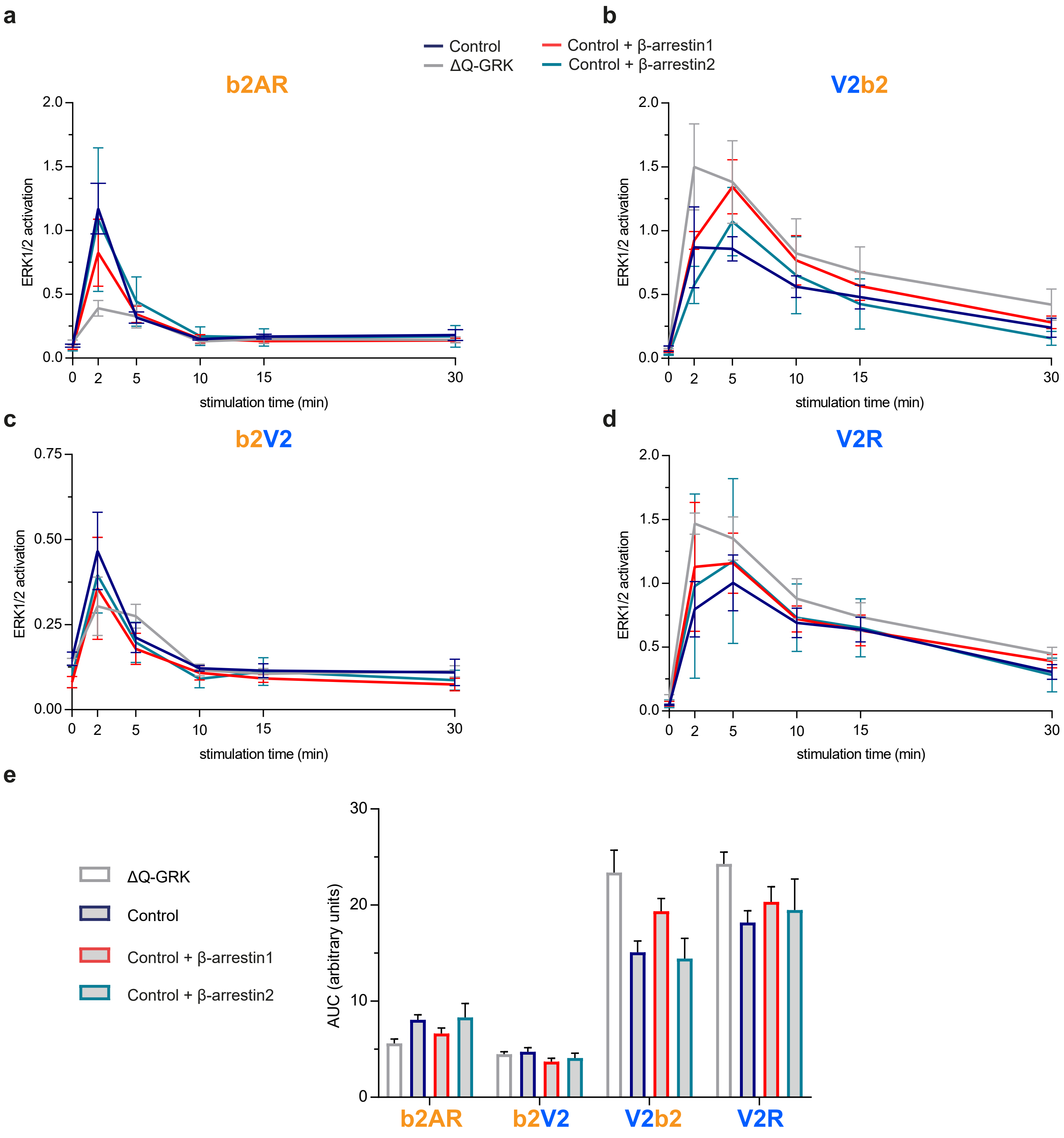

Supplementary Figure 4: Overexpression of β-arrestin enhances ERK1/2 phosphorylation. a-d, Western blots detecting phosphorylated ERK (pERK) and total ERK over time in Control or ΔQ-GRK cells, stably expressing b2AR (**a**), V2b2 (**b**), b2V2 (**c**) or V2R (**d**), of *n=3* independent experiments were quantified. Data are normalized to the respective basal levels of pERK and are shown as fold change ERK1/2 phosphorylation over time ± SEM. Measurements in Control and ΔQ-GRK cells under endogenous expression of β-arrestins from **Fig. 3e-h** are shown again for each receptor construct to enable direct comparison. **e,** As displayed in **Fig. 3e-h**, the area under the curve (AUC) was quantified for each condition to compare the pERK changes over time, shown as a bar graph. Statistical significance was analyzed using a 2-way ANOVA. Complete results of the statistical analysis can be accessed in **Suppl. Tab. 1**.

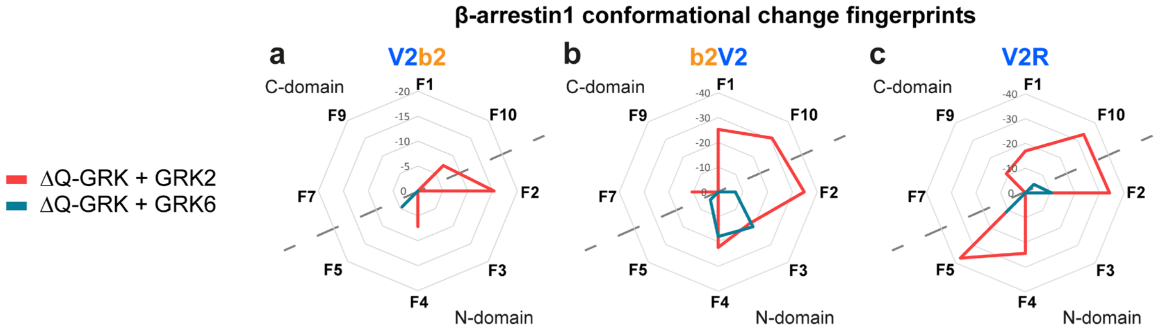

Supplementary Figure 5: Differential GPCR phosphorylation by GRK2 and GRK6 induces distinct receptor-specific β-arrestin1 conformational changes. a-c, Fingerprint of β-arrestin1 conformational change sensors measured in ΔQ-GRK cells, individually overexpressing GRK2 or GRK6, in presence of untagged V2b2 (**a**), b2V2 (**b**) or V2R (**c**). The Δ net BRET changes at 10 µM Iso (**b**) or 3 µM AVP (**a, c**) are shown as Δ net BRET change (%) of at least *n=3* independent experiments as radar plots, analogously to **Fig. 4i-l**. Sensor conditions, which did not fulfill the set pharmacological parameters (Hill slope and EC_{50} analysis as described in methods) were classified as non-responding conditions and assigned zero.

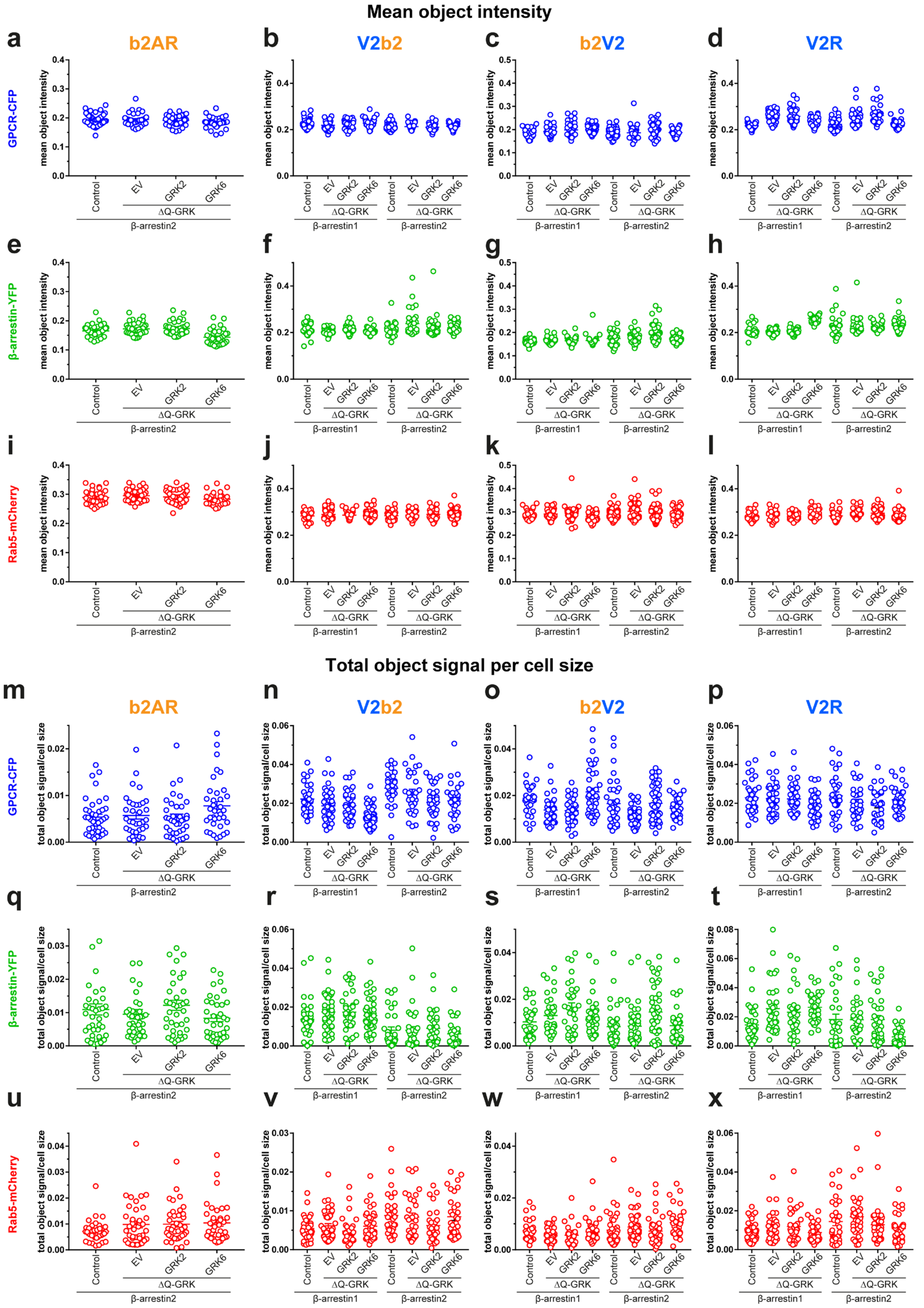

Supplementary Figure 6: Overview of mean object intensity and total object signal per cell size for each microscopy condition. Squassh segmentation parameters (mean object intensity (**a-l**) and total object per cell size (**m-x**)) for all non-stimulated confocal images that were subjected to co-localization analysis, separated by channels (GPCR-CFP (blue), β-arrestin-YFP (green), Rab5-mCherry (red)).

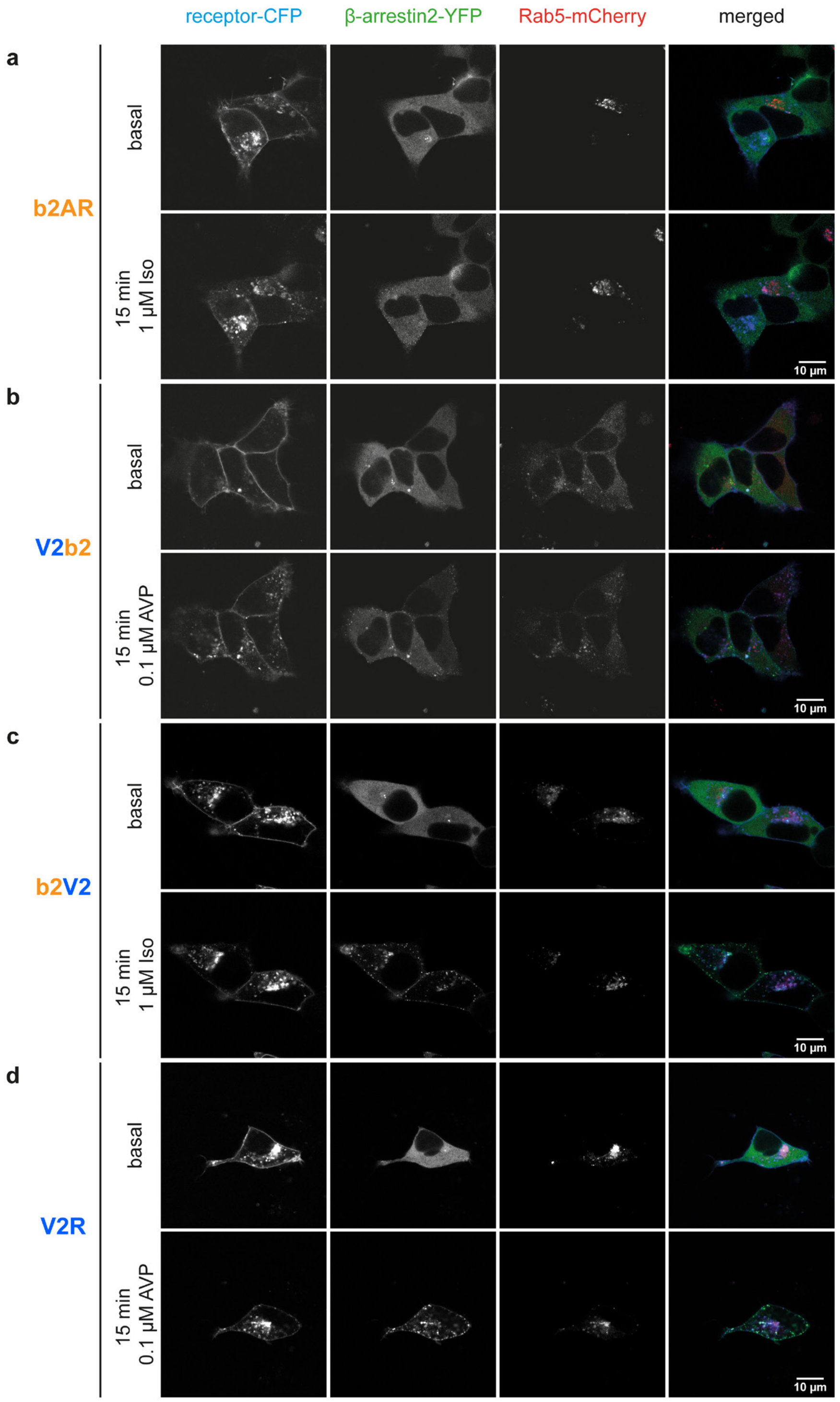

Supplementary Figure 7: Single channel images show β-arrestin translocation to early endosomes depends on the receptor C-terminus. a-d, Control cells were transfected with the indicated receptor-CFP (blue), β-arrestin-YFP (green) and early endosome marker Rab5-mCherry (red). Confocal images were taken before (basal) and after 15 min stimulation with the specified ligand (stimulated). Here, single channel and merged images are displayed for all receptors of the representative Control cell images shown in **Fig. 5a**.

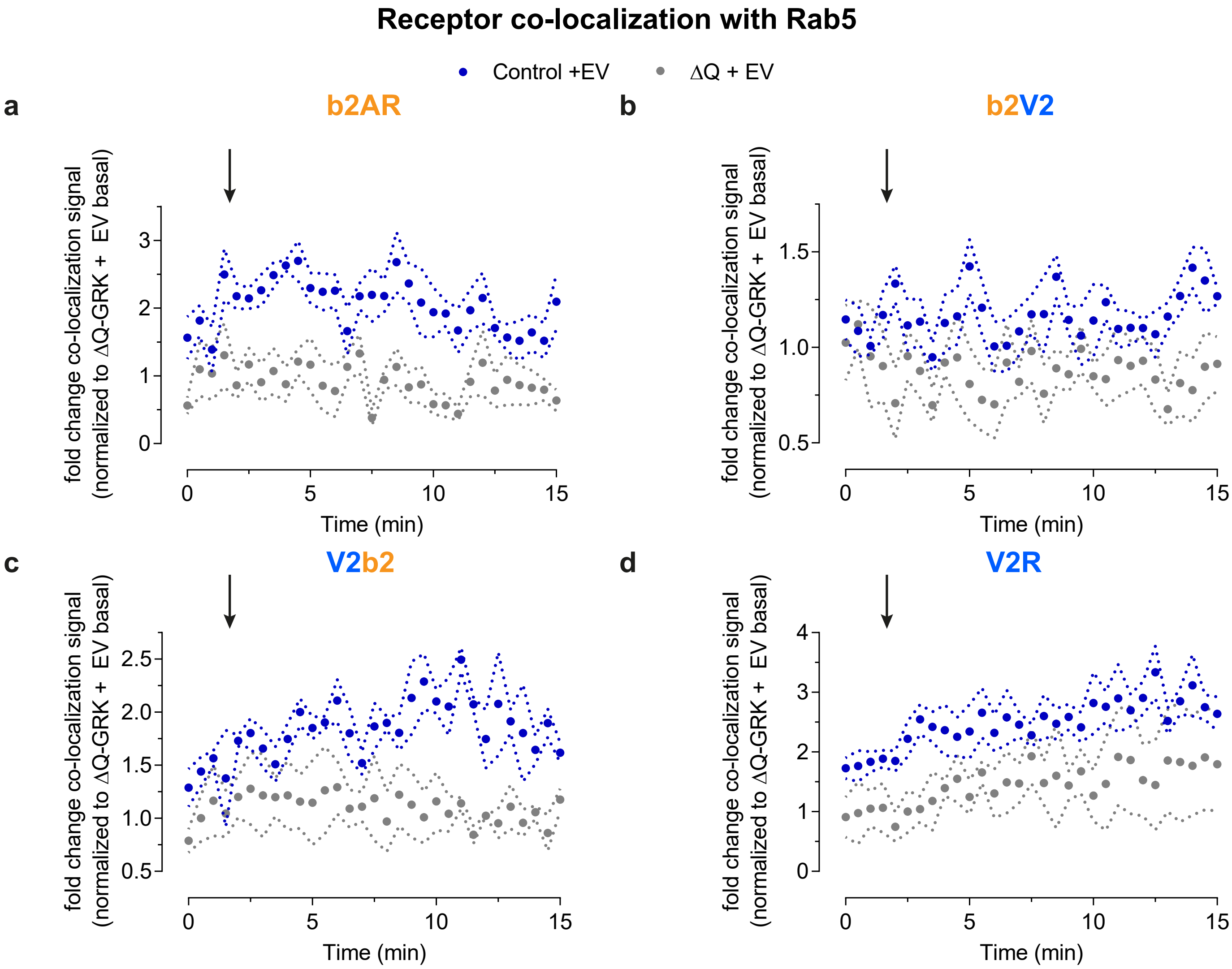

Supplementary Figure 8: Quantification of receptor and β-arrestin translocation to early endosomes over time. a-d, Control or ΔQ-GRK cells were transfected with the indicated receptor-CFP, β-arrestin2-YFP and early endosome marker Rab5-mCherry. Confocal movies were recorded over 15min with an image taken every 30 sec and ligand addition after the fourth frame (indicated by black arrow). The co-localization (signal) of the receptor with Rab5 in presence of β-arrestin2 was quantified using Squassh and SquasshAnalyst44,45, analogously to the described image analysis. For all conditions, data of at least three independent movies per condition are shown as mean fold change in co-localization (signal) ± SEM, normalized to the respective unstimulated (basal) ΔQGRK + EV condition. Cells expressing b2AR core receptor (a, b) were stimulated with 1 μM Iso, cells expressing receptors including the V2R core (c,d) were stimulated with 0.1 μM AVP.

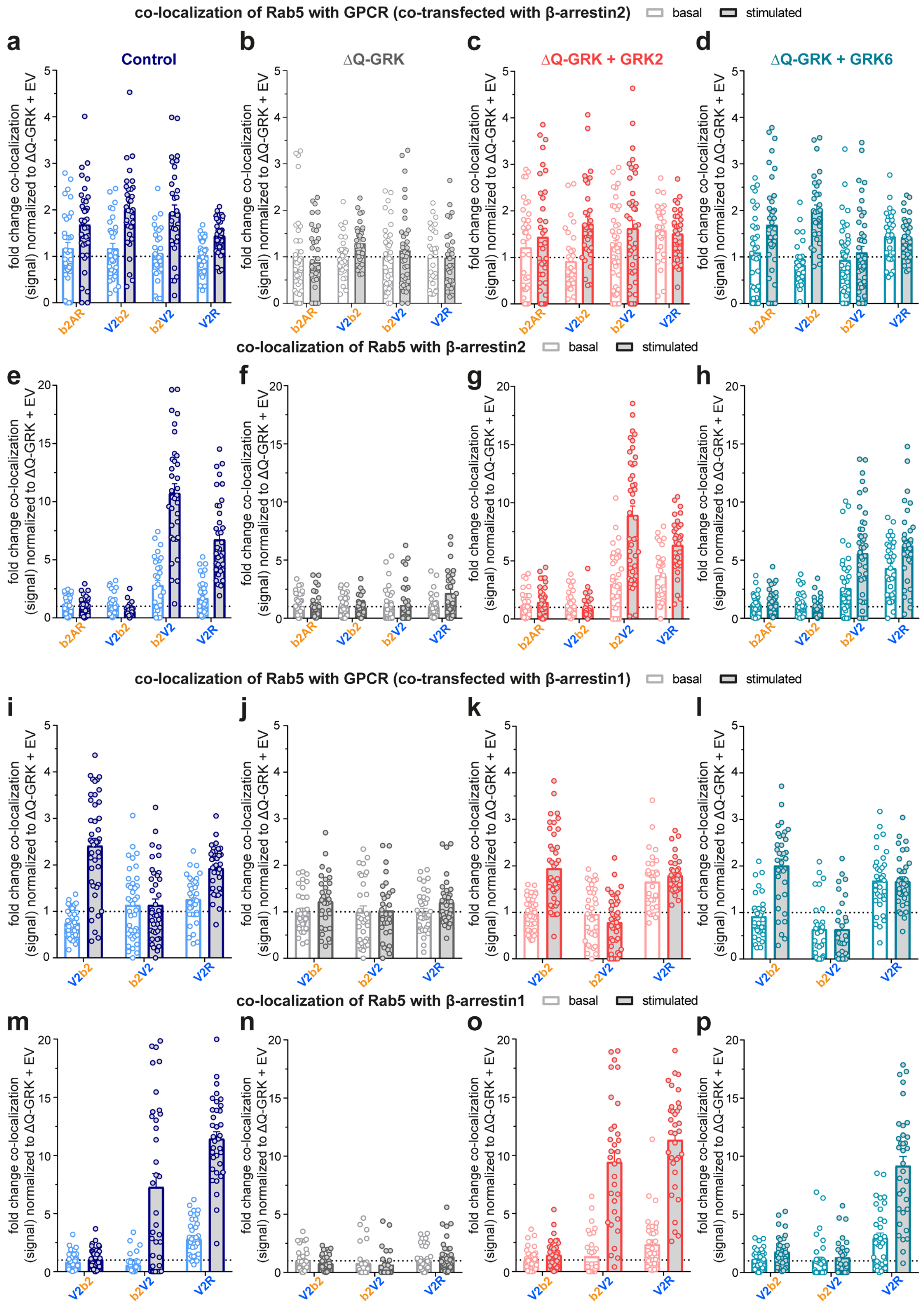

Supplementary Figure 9: Quantification of receptor and β-arrestin translocation to early endosomes. a-h, Control or ΔQ-GRK cells were transfected with the indicated receptor-CFP, β-arrestin2-YFP and early endosome marker Rab5-mCherry, as well as GRK2 or GRK6 in ΔQ-GRK cells, as indicated. Confocal images were taken before (basal; lighter color) and after 15 min of stimulation with the specified ligand (stimulated; darker color). The co-localization (signal) of the receptor with Rab5 in presence of β-arrestin2 (**a-d**) or of β-arrestin2 with Rab 5 for

each receptor construct (**e-h**) was quantified using Squassh and SquasshAnalyst[44,45]. The co-localization of Rab5 with β-arrestin2 in Control cells (**Fig. 5b**) is displayed again (**e**) to allow a direct comparison between the measured GRK conditions. **i-p,** Analogously to the experiments performed with β-arrestin2, co-localization with Rab5 was also investigated in presence of β-arrestin1-YFP for V2b2, b2V2 and V2R. For all conditions, data of at least 28 images per condition are shown as mean fold change in co-localization (signal) + SEM, normalized to the respective unstimulated (basal) ΔQ-GRK + EV condition. Statistical comparison between basal and stimulated values was performed using a two-way ANOVA, followed by a Sidak's test. Basal and stimulated values between different conditions were compared with a two-way ANOVA, followed by a Tukey's test. Detailed results can be accessed in **Suppl. Tab. 3-6**.

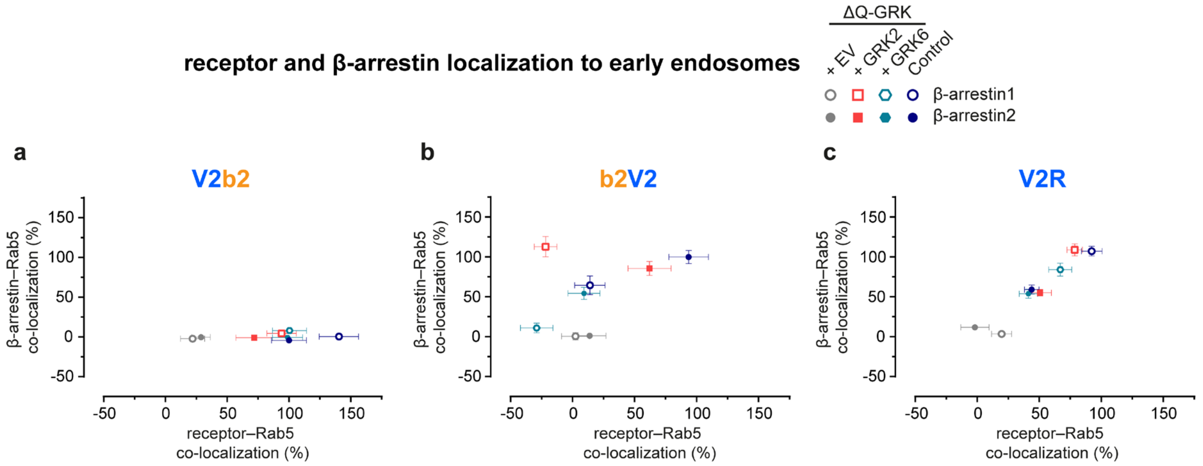

Supplementary Figure 10: β-arrestin1 translocation to early endosomes is controlled by the receptor C-terminus. a-c, Analogous to the data shown in **Fig. 5d-f**, the fold change in co-localization (signal) was analyzed for the receptor or β-arrestin1 with Rab5, as fold change over the respective basal ΔQ-GRK + EV condition for each tested receptor (V2b2 (**a**), b2V2 (**b**), V2R (**c**)) and GRK condition (Control, ΔQ-GRK +EV, +GRK2, +GRK6). The stimulated values for receptor–Rab5 co-localization are show on the x-axis and for β-arrestin1–Rab5 co-localization on the y-axis. For both dimension, the data points were normalized to the respective maximum in presence of β-arrestin2 (for receptor–Rab5 co-localization: V2b2 in Control cells, for β-arrestin2–Rab5 co-localization: b2V2 in Control cells) and are shown in percent. Data for β-arrestin2 (**Fig. 5c-f**) are shown again to allow a direct comparison. Detailed statistical analysis can be accessed in **Suppl. Tab. 4-6**.

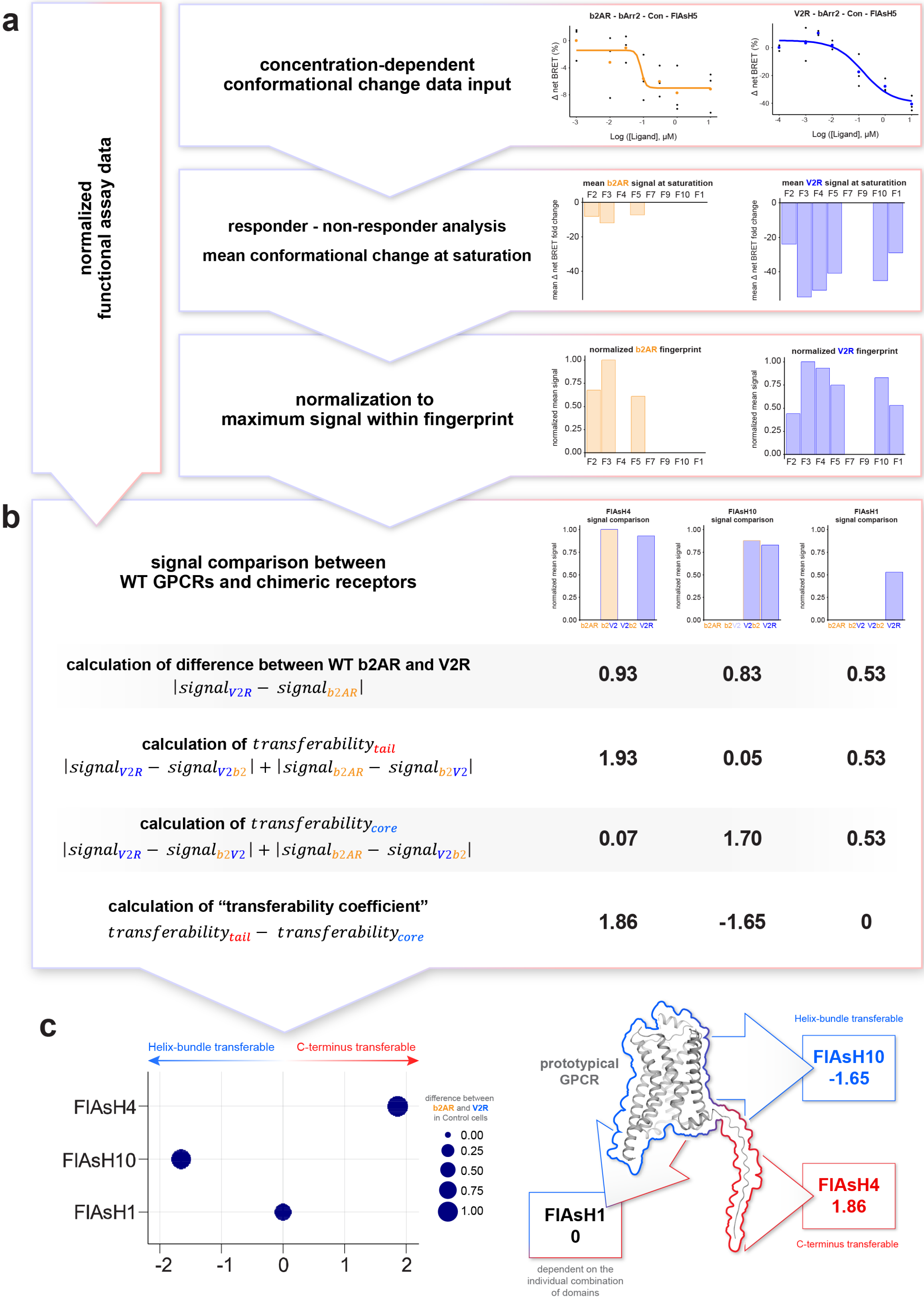

Supplementary Figure 11: Influence of helix-bundle or C-terminus on β-arrestin conformational change signals, calculated via “transferability coefficients”. a, Flowchart indicating normalization steps of input β-arrestin conformational change and functional assay data (featuring example data on the right). **b,** Calculation of “transferability coefficients” using normalized β-arrestin conformational change signals and normalized functional assay data at saturating ligand concentrations for all four receptor specific conditions (b2AR, b2V2, V2b2 and V2R,

featuring example data for β-arrestin conformational changes on the right). First, the difference between WT GPCR-induced signals was calculated, followed by the assessment of differences between receptor-specific conditions that share the same helix-bundle ($transferability_{tail}$) and C-terminus ($transferability_{core}$). Finally, subtraction of calculated $transferability_{tail}$ and $transferability_{core}$ values yields unitless transferability coefficients. A negative coefficient indicates a more pronounced influence of the helix-bundle, whereas a positive coefficient is interpreted as the GPCR C-terminus having a more pronounced influence on the molecular movement of individual β-arrestin conformational change biosensors. A value of zero suggests that the receptor-specific signals depend on the individual combination of domains. **c,** Calculated transferability coefficients of β-arrestin2-F1, F4 and F10 biosensors in Control cells (used as example data above) are plotted to indicate the influence of individual GPCR domains on conformational changes of these specific β-arrestin2 moieties. The difference in signal between WT GPCRs is shown as the size of the plotted symbols.

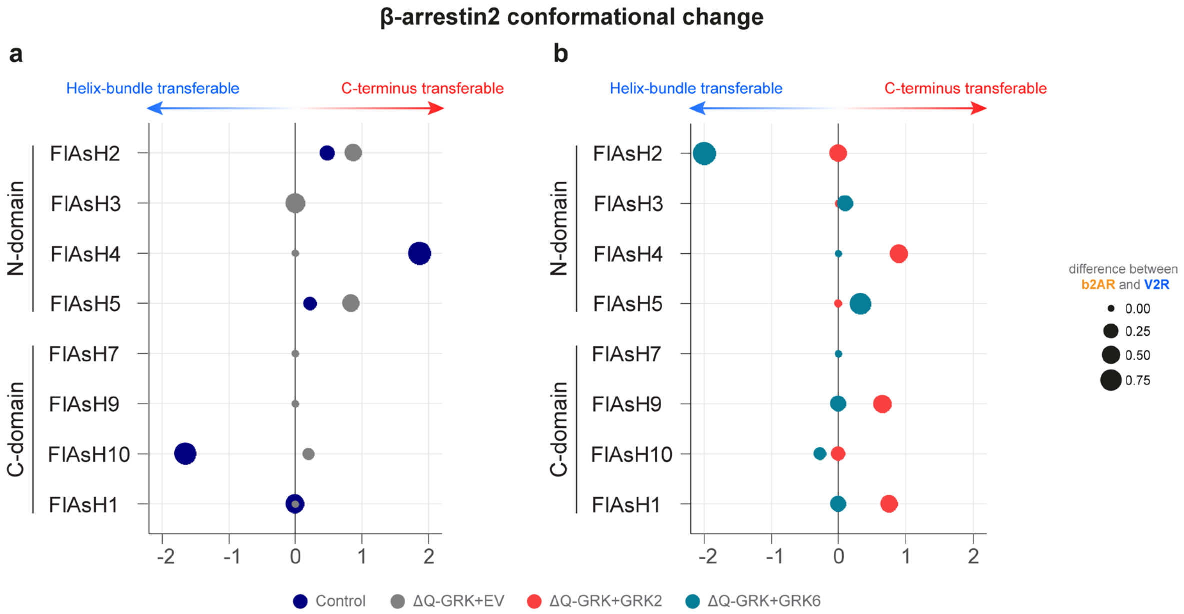

Supplementary Figure 12: "Transferability coefficents" of GRK-specific β-arrestin2 conformational changes. a, b, Calculated transferability coefficients of β-arrestin2 biosensors in Control cells or EV-transfected ΔQ-GRK cells (**a**) and under GRK2 or 6 overexpression (**b**) are plotted to indicate the influence of individual GPCR domains on conformational changes. The difference in signal between WT GPCRs is shown as the size of the plotted symbols. Calculation of these coefficients is explained in **Suppl. Fig. 11**.

Supplementary Table 1: Detailed statistical results of the analysis of the area under the curve (AUC), derived from Western blots, shown in **Fig. 3e-h** and **Suppl. Fig. 4e**. AUC of fold change of ERK1/2 phosphorylation over time was compared between Control and ΔQ-GRK cells, stably expressing the b2AR, V2b2, b2V2 or V2R, using an unpaired, two-tailed t-test (**Fig. 3e-h**) or between Control cells in absence or presence of transient overexpression of β-arrestin1 or β-arrestin2 with a two-way ANOVA, followed by a Dunnett's test (**Suppl. Fig. 4e**). For each condition the mean difference, 95% confidence interval (CI) of the difference, and the adjusted *p* value are shown (*ns,* not significant; *, $p < 0.05$; **, $p < 0.01$; ***, $p < 0.001$; ****, $p < 0.0001$).

**conditions**				
**AUC of pERK over time**	**unpaired two-tailed t-test**			
Control vs ΔQ-GRK	**diff. betw. means**	**95% CI**	***p* value**	
b2AR	2.463 ± 0.6844	0.5629 to 4.363	0.0228	*
V2b2	-8.3 ± 2.603	-15.53 to -1.072	0.0333	*
b2V2	0.257 ± 0.4894	-1.102 to 1.616	0.6272	ns
V2R	-6.11 ± 1.734	-10.92 to -1.296	0.0244	*
**AUC of pERK over time**	**two-way ANOVA, followed by a Dunnetts's test**			
Control cells + EV vs. + β-arrestin1	**mean diff.**	**95% CI of diff.**	**adjusted *p* value**	
b2AR	1.428	-1.964 to 4.82	0.4888	ns
V2b2	-4.27	-13.75 to 5.205	0.4457	ns
b2V2	1.040	-0.65 to 2.73	0.2507	ns
V2R	-2.16	-13.59 to 9.27	0.8528	ns
Control cells + EV vs. + β-arrestin2				
b2AR	-0.234	-3.626 to 3.158	0.9784	ns
V2b2	0.65	-8.825 to 10.13	0.9787	ns
b2V2	0.642	-1.048 to 2.332	0.5517	ns
V2R	-1.31	-12.74 to 10.12	0.942	ns

Supplementary Table 2: Detailed statistical results of the analysis of the normalized OD 405 nm (%) for the proximal and distal phosphorylation cluster in ΔQ-GRK cells stably overexpressing one of the investigated receptors and GRK2 or 6 in comparison to the respective Control cells (**Fig. 4a-h**). Values of maximal ligand concentration and vehicle stimulation were compared using a two-way ANOVA, followed by a Tukey's test. For each condition the mean difference, 95% confidence interval (CI) of the difference, and the adjusted *p* value are shown (*ns,* not significant; *, $p < 0.05$; **, $p < 0.01$; ***, $p < 0.001$; ****, $p < 0.0001$).

conditions	two-way ANOVA, followed by Tukey's test			
normalized OD 405 nm				
b2AR - proximal phosphorylation	mean diff.	95% CI of diff.	adjusted *p* value	
vehicle stimulation				
Con vs. GRK2	-4.938	-13.99 to 4.115	0.3761	ns
Con vs. GRK6	-16.3	-25.35 to -7.245	0.0004	***
GRK2 vs. GRK6	-11.36	-20.41 to -2.307	0.0121	*
maximal stimulation				
Con vs. GRK2	59.7	50.65 to 68.76	<0.0001	****
Con vs. GRK6	-7.346	-16.4 to 1.707	0.1275	ns
GRK2 vs. GRK6	-67.05	-76.1 to -58	<0.0001	****
b2AR - distal phosphorylation				
vehicle stimulation				
Con vs. GRK2	-1.796	-19.61 to 16.02	0.9657	ns
Con vs. GRK6	-5.46	-23.28 to 12.36	0.7275	ns
GRK2 vs. GRK6	-3.664	-21.48 to 14.15	0.8655	ns
maximal stimulation				
Con vs. GRK2	30.1	12.28 to 47.92	0.0009	***
Con vs. GRK6	-2.344	-20.16 to 15.47	0.9424	ns
GRK2 vs. GRK6	-32.44	-50.26 to -14.62	0.0004	***
V2b2 - proximal phosphorylation				
vehicle stimulation				
Con vs. GRK2	-0.408	-13.11 to 12.29	0.9965	ns
Con vs. GRK6	-11.48	-24.18 to 1.22	0.0818	ns
GRK2 vs. GRK6	-11.07	-23.78 to 1.628	0.0957	ns
maximal stimulation				
Con vs. GRK2	64.07	51.36 to 76.77	<0.0001	****
Con vs. GRK6	32	19.3 to 44.7	<0.0001	****
GRK2 vs. GRK6	-32.07	-44.77 to -19.37	<0.0001	****
V2b2 - distal phosphorylation				
vehicle stimulation				
Con vs. GRK2	0.122	-8.521 to 8.765	0.9993	ns
Con vs. GRK6	-5.258	-13.9 to 3.385	0.2999	ns
GRK2 vs. GRK6	-5.38	-14.02 to 3.263	0.2843	ns
maximal stimulation				
Con vs. GRK2	55.16	46.52 to 63.8	<0.0001	****
Con vs. GRK6	42.98	34.34 to 51.62	<0.0001	****
GRK2 vs. GRK6	-12.18	-20.83 to -3.541	0.0048	**
b2V2 - proximal phosphorylation				
vehicle stimulation				
Con vs. GRK2	-1.938	-21.66 to 17.78	0.9674	ns
Con vs. GRK6	-5.334	-25.06 to 14.39	0.7799	ns
GRK2 vs. GRK6	-3.396	-23.12 to 16.33	0.9035	ns
maximal stimulation				
Con vs. GRK2	59.28	39.56 to 79.01	<0.0001	****
Con vs. GRK6	12.46	-7.267 to 32.18	0.2746	ns
GRK2 vs. GRK6	-46.83	-66.55 to -27.11	<0.0001	****
b2V2 - distal phosphorylation				
vehicle stimulation				
Con vs. GRK2	-4.404	-29.52 to 20.71	0.9001	ns
Con vs. GRK6	-16.05	-41.16 to 9.061	0.2666	ns
GRK2 vs. GRK6	-11.65	-36.76 to 13.46	0.4888	ns
maximal stimulation				
Con vs. GRK2	59.66	34.54 to 84.77	<0.0001	****
Con vs. GRK6	1.434	-23.68 to 26.55	0.9889	ns
GRK2 vs. GRK6	-58.22	-83.33 to -33.11	<0.0001	****
V2R - proximal phosphorylation				
vehicle stimulation				
Con vs. GRK2	1.108	-11.58 to 13.8	0.9742	ns
Con vs. GRK6	-13.25	-25.94 to -0.5594	0.0395	*
GRK2 vs. GRK6	-14.36	-27.05 to -1.667	0.0245	*
maximal stimulation				
Con vs. GRK2	43.88	31.19 to 56.57	<0.0001	****
Con vs. GRK6	17.67	4.975 to 30.36	0.0053	**
GRK2 vs. GRK6	-26.22	-38.91 to -13.53	<0.0001	****
V2R - distal phosphorylation				
vehicle stimulation				
Con vs. GRK2	0.058	-15.67 to 15.79	>0.9999	ns
Con vs. GRK6	-12.16	-27.89 to 3.567	0.1518	ns
GRK2 vs. GRK6	-12.22	-27.95 to 3.509	0.1493	ns
maximal stimulation				
Con vs. GRK2	42.96	27.23 to 58.69	<0.0001	****
Con vs. GRK6	26.76	11.03 to 42.49	0.0008	***
GRK2 vs. GRK6	-16.2	-31.93 to -0.4653	0.0427	*

Supplementary Table 3: Detailed statistical results of the analysis of the microscopy co-localization quantification, shown in **Fig. 5b** (for co-localization of Rab5 with β-arrestin2 in Control cells) and **Suppl. Fig. 9**. Here, basal and stimulated values of co-localization for each tested receptor with Rab5, in presence of β-arrestin2 or β-arrestin1, as well as for β-arrestin2 or β-arrestin1 with Rab5 were compared using a two-way ANOVA, followed by a Sidak's test (*ns,* not significant; *, $p < 0.05$; **, $p < 0.01$; ***, $p < 0.001$; ****, $p < 0.0001$). For each condition the mean difference, 95% confidence interval (CI) of the difference, and the adjusted *p* value are shown.

conditions	two-way ANOVA, followed by Sidak's test			
co-localization of Rab5 with GPCR (β-arrestin2)				
basal vs. stim in Control	mean diff.	95% CI of diff.	adjusted *p* value	
b2AR	-0.5116	-0.923 to -0.1001	0.0081	**
V2b2	-0.8347	-1.264 to -0.4051	<0.0001	****
b2V2	-0.8738	-1.311 to -0.4367	<0.0001	****
V2R	-0.4242	-0.8384 to -0.009991	0.0424	*
basal vs. stim in ΔQ-GRK				
b2AR	0.1144	-0.2837 to 0.5125	0.9222	ns
V2b2	-0.2891	-0.7269 to 0.1487	0.3406	ns
b2V2	-0.1381	-0.5362 to 0.2599	0.8568	ns
V2R	0.01865	-0.4343 to 0.4716	>0.9999	ns
basal vs. stim in ΔQ-GRK + GRK2				
b2AR	-0.225	-0.7439 to 0.2939	0.7281	ns
V2b2	-0.8062	-1.352 to -0.2605	0.001	**
b2V2	-0.3911	-0.8749 to 0.09272	0.1635	ns
V2R	0.07107	-0.4831 to 0.6252	0.996	ns
basal vs. stim in ΔQ-GRK cells				
b2AR	-0.5955	-1.029 to -0.1621	0.0026	**
V2b2	-1.176	-1.635 to -0.7174	<0.0001	****
b2V2	-0.1591	-0.5491 to 0.231	0.77	ns
V2R	0.02669	-0.422 to 0.4754	0.9998	ns
co-localization of Rab5 with GPCR (β-arrestin1)				
basal vs. stim in Control				
V2b2	-1.656	-2.021 to -1.291	<0.0001	****
b2V2	-0.00115	-0.3824 to 0.3801	>0.9999	ns
V2R	-0.6655	-1.077 to -0.2545	0.0004	***
basal vs. stim in ΔQ-GRK				
V2b2	-0.2202	-0.5477 to 0.1073	0.2879	ns
b2V2	-0.02428	-0.3622 to 0.3136	0.9974	ns
V2R	-0.1975	-0.5155 to 0.1206	0.3559	ns
basal vs. stim in ΔQ-GRK + GRK2				
V2b2	-0.9736	-1.274 to -0.6735	<0.0001	****
b2V2	0.1774	-0.1409 to 0.4956	0.4512	ns
V2R	-0.1337	-0.4584 to 0.191	0.6897	ns
basal vs. stim in ΔQ-GRK cells				
V2b2	-1.094	-1.467 to -0.7197	<0.0001	****
b2V2	-0.001348	-0.4026 to 0.3999	>0.9999	ns
V2R	0.009035	-0.3647 to 0.3828	>0.9999	ns
co-localization of Rab5 with β-arrestin2				
basal vs. stim in Control				
b2AR	0.1515	-1.2 to 1.503	0.9976	ns
V2b2	0.4558	-0.9749 to 1.887	0.8907	ns
b2V2	-7.924	-9.367 to -6.481	<0.0001	****
V2R	-5.039	-6.39 to -3.688	<0.0001	****
basal vs. stim in ΔQ-GRK				
b2AR	0.05554	-0.6855 to 0.7966	0.9995	ns
V2b2	0.02879	-0.7982 to 0.8558	>0.9999	ns
b2V2	-0.1135	-0.8645 to 0.6375	0.9924	ns
V2R	-1.147	-1.988 to -0.3063	0.0029	**
basal vs. stim in ΔQ-GRK + GRK2				
b2AR	-0.2852	-1.753 to 1.182	0.9805	ns
V2b2	0.245	-1.352 to 1.842	0.992	ns
b2V2	-5.928	-7.35 to -4.506	<0.0001	****
V2R	-2.64	-4.277 to -1.004	0.0003	***
basal vs. stim in ΔQ-GRK cells				
b2AR	-0.2084	-1.58 to 1.163	0.9923	ns
V2b2	0.3268	-1.159 to 1.812	0.9694	ns
b2V2	-2.999	-4.296 to -1.702	<0.0001	****
V2R	-1.933	-3.374 to -0.4926	0.0035	**
co-localization of Rab5 with β-arrestin1				
basal vs. stim in Control				
V2b2	-0.2404	-1.881 to 1.401	0.9792	ns
b2V2	-6.711	-8.491 to -4.931	<0.0001	****
V2R	-8.609	-10.44 to -6.779	<0.0001	****
basal vs. stim in ΔQ-GRK				
V2b2	0.222	-0.4559 to 0.9	0.8159	ns
b2V2	0.1382	-0.5789 to 0.8554	0.9545	ns
V2R	-0.3441	-1.002 to 0.3142	0.506	ns
basal vs. stim in ΔQ-GRK + GRK2				
V2b2	-0.4809	-2.049 to 1.087	0.8436	ns
b2V2	-8.121	-9.868 to -6.375	<0.0001	****
V2R	-8.982	-10.7 to -7.263	<0.0001	****
basal vs. stim in ΔQ-GRK cells				
V2b2	-0.64	-2.069 to 0.7888	0.6299	ns
b2V2	-0.2647	-1.773 to 1.243	0.965	ns
V2R	-6.207	-7.656 to -4.757	<0.0001	****

Supplementary Table 4: Detailed statistical results of the analysis of the microscopy co-localization, shown in **Suppl. Fig. 9**. Here, basal or stimulated values of co-localization with Rab were compared between tested receptors, in presence of β-arrestin2, using a two-way ANOVA, followe by a Tukey's test (*ns,* not significant; *, $p < 0.05$; **, $p < 0.01$; ***, $p < 0.001$; ****, $p < 0.0001$). For eac condition the mean difference, 95% confidence interval (CI) of the difference, and the adjusted *p* value are shown.

**conditions**	**two-way ANOVA, followed by Tukey's test**			
**co-localization of Rab5 with GPCR (β-arrestin2)**				
basal conditions in Control	**mean diff.**	**95% CI of diff.**	**adjusted *p* value**	
b2AR vs. V2b2	-0.00006492	-0.4365 to 0.4364	>0.9999	ns
b2AR vs. b2V2	0.103	-0.3446 to 0.5505	0.9336	ns
b2AR vs. V2R	0.1516	-0.2784 to 0.5816	0.7989	ns
V2b2 vs. b2V2	0.103	-0.3508 to 0.5568	0.936	ns
V2b2 vs. V2R	0.1516	-0.2848 to 0.5881	0.8058	ns
b2V2 vs. V2R	0.04861	-0.3989 to 0.4962	0.9923	ns
stimulated conditions in Control				
b2AR vs. V2b2	-0.3232	-0.7539 to 0.1075	0.2139	ns
b2AR vs. b2V2	-0.2593	-0.6866 to 0.1681	0.3983	ns
b2AR vs. V2R	0.2389	-0.1822 to 0.66	0.459	ns
V2b2 vs. b2V2	0.06389	-0.3757 to 0.5035	0.9819	ns
V2b2 vs. V2R	0.5621	0.1286 to 0.9956	0.0051	**
b2V2 vs. V2R	0.4982	0.06804 to 0.9284	0.0158	*
basal conditions in ΔQ-GRK				
b2AR vs. V2b2	-0.0000000007	-0.4326 to 0.4326	>0.9999	ns
b2AR vs. b2V2	0.0000000002	-0.4131 to 0.4131	>0.9999	ns
b2AR vs. V2R	-0.0000000004	-0.4451 to 0.4451	>0.9999	ns
V2b2 vs. b2V2	0.0000000009	-0.4326 to 0.4326	>0.9999	ns
V2b2 vs. V2R	0.0000000003	-0.4633 to 0.4633	>0.9999	ns
b2V2 vs. V2R	-0.0000000007	-0.4451 to 0.4451	>0.9999	ns
stimulated conditions in ΔQ-GRK				
b2AR vs. V2b2	-0.4035	-0.8335 to 0.02658	0.0748	ns
b2AR vs. b2V2	-0.2525	-0.6602 to 0.1551	0.3795	ns
b2AR vs. V2R	-0.09571	-0.5297 to 0.3383	0.9409	ns
V2b2 vs. b2V2	0.1509	-0.2791 to 0.581	0.8008	ns
V2b2 vs. V2R	0.3078	-0.1473 to 0.7628	0.3008	ns
b2V2 vs. V2R	0.1568	-0.2772 to 0.5908	0.7865	ns
basal conditions in ΔQ-GRK + GRK2				
b2AR vs. V2b2	0.2988	-0.2519 to 0.8495	0.4989	ns
b2AR vs. b2V2	-0.01804	-0.5399 to 0.5039	0.9997	ns
b2AR vs. V2R	-0.3651	-0.9203 to 0.1901	0.3257	ns
V2b2 vs. b2V2	-0.3169	-0.8512 to 0.2175	0.4195	ns
V2b2 vs. V2R	-0.6639	-1.231 to -0.097	0.0143	*
b2V2 vs. V2R	-0.3471	-0.8861 to 0.1919	0.3447	ns
stimulated conditions in ΔQ-GRK + GRK2				
b2AR vs. V2b2	-0.2824	-0.8295 to 0.2647	0.5421	ns
b2AR vs. b2V2	-0.1841	-0.6965 to 0.3283	0.7894	ns
b2AR vs. V2R	-0.06903	-0.6206 to 0.4826	0.9883	ns
V2b2 vs. b2V2	0.09824	-0.4306 to 0.6271	0.9634	ns
V2b2 vs. V2R	0.2133	-0.3536 to 0.7803	0.7651	ns
b2V2 vs. V2R	0.1151	-0.4184 to 0.6486	0.9444	ns
basal conditions in ΔQ-GRK + GRK6				
b2AR vs. V2b2	0.2734	-0.1931 to 0.7399	0.43	ns
b2AR vs. b2V2	0.1553	-0.2746 to 0.5851	0.7869	ns
b2AR vs. V2R	-0.3518	-0.8111 to 0.1074	0.1981	ns
V2b2 vs. b2V2	-0.1182	-0.5592 to 0.3229	0.9	ns
V2b2 vs. V2R	-0.6252	-1.095 to -0.1555	0.0037	**
b2V2 vs. V2R	-0.5071	-0.9404 to -0.07369	0.0144	*
stimulated conditions in ΔQ-GRK + GRK6				
b2AR vs. V2b2	-0.3074	-0.7612 to 0.1464	0.2998	ns
b2AR vs. b2V2	0.5917	0.1716 to 1.012	0.0018	**
b2AR vs. V2R	0.2704	-0.1798 to 0.7206	0.4079	ns
V2b2 vs. b2V2	0.8991	0.462 to 1.336	<0.0001	****
V2b2 vs. V2R	0.5778	0.1117 to 1.044	0.0082	**
b2V2 vs. V2R	-0.3213	-0.7547 to 0.1121	0.2236	ns

Supplementary Table 5: Detailed statistical results of the analysis of the microscopy co-localization quantification, shown in **Fig. 5b** (for co-localization of Rab5 with β-arrestin2 in Control cells) and **Suppl. Fig. 9**. Here, basal or stimulated values of β-arrestin2 co-localization with Rab5 were compared between tested receptors, using a two-way ANOVA, followed by a Tukey's test (*ns,* not significant; *, *p* < 0.05; **, *p* < 0.01; ***, *p* < 0.001; ****, *p* < 0.0001). For each condition the mean difference, 95% confidence interval (CI) of the difference, and the adjusted *p* value are shown.

**conditions**	**two-way ANOVA, followed by Tukey's test**			
**co-localization of Rab5 with β-arrestin2**				
basal conditions in Control	**mean diff.**	**95% CI of diff.**	**adjusted *p* value**	
b2AR vs. V2b2	-0.04462	-1.479 to 1.39	0.9998	ns
b2AR vs. b2V2	-1.811	-3.27 to -0.3522	0.0081	**
b2AR vs. V2R	-0.707	-2.1 to 0.6859	0.556	ns
V2b2 vs. b2V2	-1.766	-3.265 to -0.2678	0.0135	*
V2b2 vs. V2R	-0.6624	-2.097 to 0.7721	0.6314	ns
b2V2 vs. V2R	1.104	-0.3549 to 2.563	0.2074	ns
stimulated conditions in Control				
b2AR vs. V2b2	0.2598	-1.175 to 1.694	0.966	ns
b2AR vs. b2V2	-9.887	-11.31 to -8.463	<0.0001	****
b2AR vs. V2R	-5.898	-7.29 to -4.505	<0.0001	****
V2b2 vs. b2V2	-10.15	-11.61 to -8.682	<0.0001	****
V2b2 vs. V2R	-6.157	-7.592 to -4.723	<0.0001	****
b2V2 vs. V2R	3.989	2.566 to 5.412	<0.0001	****
basal conditions in ΔQ-GRK				
b2AR vs. V2b2	-0.00000000027	-0.8105 to 0.8105	>0.9999	ns
b2AR vs. b2V2	-0.00000000027	-0.7793 to 0.7793	>0.9999	ns
b2AR vs. V2R	-0.00000000027	-0.8256 to 0.8256	>0.9999	ns
V2b2 vs. b2V2	0	-0.8156 to 0.8156	>0.9999	ns
V2b2 vs. V2R	0	-0.86 to 0.86	>0.9999	ns
b2V2 vs. V2R	0	-0.8306 to 0.8306	>0.9999	ns
stimulated conditions in ΔQ-GRK				
b2AR vs. V2b2	-0.02676	-0.8351 to 0.7816	0.9998	ns
b2AR vs. b2V2	-0.169	-0.9278 to 0.5897	0.9392	ns
b2AR vs. V2R	-1.203	-2.011 to -0.3944	0.0009	***
V2b2 vs. b2V2	-0.1423	-0.9553 to 0.6707	0.9691	ns
V2b2 vs. V2R	-1.176	-2.036 to -0.3165	0.0027	**
b2V2 vs. V2R	-1.034	-1.847 to -0.2207	0.0063	**
basal conditions in ΔQ-GRK + GRK2				
b2AR vs. V2b2	-0.006178	-1.589 to 1.577	>0.9999	ns
b2AR vs. b2V2	-1.857	-3.35 to -0.3631	0.008	**
b2AR vs. V2R	-2.579	-4.19 to -0.9668	0.0003	***
V2b2 vs. b2V2	-1.851	-3.425 to -0.2764	0.0138	*
V2b2 vs. V2R	-2.572	-4.259 to -0.8857	0.0006	***
b2V2 vs. V2R	-0.7218	-2.325 to 0.8812	0.6501	ns
stimulated conditions in ΔQ-GRK + GRK2				
b2AR vs. V2b2	0.524	-1.055 to 2.103	0.8267	ns
b2AR vs. b2V2	-7.5	-8.986 to -6.014	<0.0001	****
b2AR vs. V2R	-4.934	-6.526 to -3.341	<0.0001	****
V2b2 vs. b2V2	-8.024	-9.567 to -6.48	<0.0001	****
V2b2 vs. V2R	-5.458	-7.104 to -3.811	<0.0001	****
b2V2 vs. V2R	2.566	1.008 to 4.123	0.0002	***
basal conditions in ΔQ-GRK + GRK6				
b2AR vs. V2b2	-0.06824	-1.559 to 1.423	0.9994	ns
b2AR vs. b2V2	-1.386	-2.774 to 0.002559	0.0506	ns
b2AR vs. V2R	-3.103	-4.557 to -1.648	<0.0001	****
V2b2 vs. b2V2	-1.318	-2.775 to 0.1397	0.0923	ns
V2b2 vs. V2R	-3.034	-4.555 to -1.514	<0.0001	****
b2V2 vs. V2R	-1.717	-3.137 to -0.2964	0.0106	*
stimulated conditions in ΔQ-GRK + GRK6				
b2AR vs. V2b2	0.467	-0.9901 to 1.924	0.841	ns
b2AR vs. b2V2	-4.176	-5.54 to -2.813	<0.0001	****
b2AR vs. V2R	-4.827	-6.273 to -3.382	<0.0001	****
V2b2 vs. b2V2	-4.643	-6.061 to -3.226	<0.0001	****
V2b2 vs. V2R	-5.294	-6.791 to -3.798	<0.0001	****
b2V2 vs. V2R	-0.6509	-2.056 to 0.7544	0.6293	ns

Supplementary Table 6: Detailed statistical results of the analysis of the microscopy co-localization quantification, shown in **Suppl. Fig. 9**. Here, basal or stimulated values of co-localization for each tested receptor with Rab5, in presence of β-arrestin1, as well as for β-arrestin1 with Rab5 were compared using a two-way ANOVA followed by a Tukey's test (*ns,* not significant; *, $p < 0.05$; **, $p < 0.01$; ***, $p < 0.001$; ****, $p < 0.0001$). For each condition the mean difference, 95% confidence interval (CI) of the difference, and the adjusted *p* value are shown.

**conditions**	**two-way ANOVA, followed by Tukey's test**			
**co-localization of Rab5 with GPCR (β-arrestin1)**				
basal conditions in Control	**mean diff.**	**95% CI of diff.**	**adjusted *p* value**	
V2b2 vs. b2V2	-0.3857	-0.7531 to -0.01835	0.0371	*
V2b2 vs. V2R	-0.5073	-0.8918 to -0.1228	0.0059	**
b2V2 vs. V2R	-0.1215	-0.5145 to 0.2714	0.7461	ns
stimulated conditions in Control				
V2b2 vs. b2V2	1.269	0.9041 to 1.634	<0.0001	****
V2b2 vs. V2R	0.4831	0.1049 to 0.8613	0.0081	**
b2V2 vs. V2R	-0.7859	-1.17 to -0.4014	<0.0001	****
basal conditions in ΔQ-GRK				
V2b2 vs. b2V2	0.0000000003	-0.3263 to 0.3263	>0.9999	ns
V2b2 vs. V2R	0.0000000006	-0.3165 to 0.3165	>0.9999	ns
b2V2 vs. V2R	0.0000000003	-0.3218 to 0.3218	>0.9999	ns
stimulated conditions in ΔQ-GRK				
V2b2 vs. b2V2	0.1959	-0.1304 to 0.5222	0.3336	ns
V2b2 vs. V2R	0.02272	-0.2938 to 0.3393	0.9843	ns
b2V2 vs. V2R	-0.1732	-0.4949 to 0.1486	0.4131	ns
basal conditions in ΔQ-GRK + GRK2				
V2b2 vs. b2V2	0.01026	-0.2981 to 0.3186	0.9966	ns
V2b2 vs. V2R	-0.6851	-0.9934 to -0.3767	<0.0001	****
b2V2 vs. V2R	-0.6953	-1.014 to -0.3769	<0.0001	****
stimulated conditions in ΔQ-GRK + GRK2				
V2b2 vs. b2V2	1.161	0.863 to 1.459	<0.0001	****
V2b2 vs. V2R	0.1548	-0.1501 to 0.4597	0.4555	ns
b2V2 vs. V2R	-1.006	-1.319 to -0.6943	<0.0001	****
basal conditions in ΔQ-GRK + GRK6				
V2b2 vs. b2V2	0.2773	-0.1073 to 0.6619	0.2067	ns
V2b2 vs. V2R	-0.7664	-1.136 to -0.3971	<0.0001	****
b2V2 vs. V2R	-1.044	-1.426 to -0.6617	<0.0001	****
stimulated conditions in ΔQ-GRK + GRK6				
V2b2 vs. b2V2	1.37	0.9935 to 1.745	<0.0001	****
V2b2 vs. V2R	0.3362	-0.02763 to 0.7001	0.0767	ns
b2V2 vs. V2R	-1.033	-1.412 to -0.6548	<0.0001	****
**co-localization of Rab5 with β-arrestin1**				
basal conditions in Control	**mean diff.**	**95% CI of diff.**	**adjusted *p* value**	
V2b2 vs. b2V2	0.243	-1.419 to 1.905	0.9365	ns
V2b2 vs. V2R	-2.015	-3.715 to -0.3153	0.0154	*
b2V2 vs. V2R	-2.258	-4.017 to -0.4999	0.0077	**
stimulated conditions in Control				
V2b2 vs. b2V2	-6.228	-7.923 to -4.532	<0.0001	****
V2b2 vs. V2R	-10.38	-12.09 to -8.675	<0.0001	****
b2V2 vs. V2R	-4.156	-5.938 to -2.374	<0.0001	****
basal conditions in ΔQ-GRK				
V2b2 vs. b2V2	0.2149	-0.4663 to 0.8961	0.7368	ns
V2b2 vs. V2R	0	-0.6552 to 0.6552	>0.9999	ns
b2V2 vs. V2R	-0.2149	-0.8868 to 0.457	0.7306	ns
stimulated conditions in ΔQ-GRK				
V2b2 vs. b2V2	0.1311	-0.5562 to 0.8185	0.8942	ns
V2b2 vs. V2R	-0.5662	-1.221 to 0.08908	0.1053	ns
b2V2 vs. V2R	-0.6973	-1.375 to -0.01918	0.0423	*
basal conditions in ΔQ-GRK + GRK2				
V2b2 vs. b2V2	-0.3451	-1.957 to 1.267	0.8688	ns
V2b2 vs. V2R	-1.386	-2.997 to 0.2261	0.1077	ns
b2V2 vs. V2R	-1.041	-2.715 to 0.6335	0.3089	ns
stimulated conditions in ΔQ-GRK + GRK2				
V2b2 vs. b2V2	-7.985	-9.629 to -6.342	<0.0001	****
V2b2 vs. V2R	-9.887	-11.5 to -8.271	<0.0001	****
b2V2 vs. V2R	-1.901	-3.626 to -0.1765	0.0267	*
basal conditions in ΔQ-GRK + GRK6				
V2b2 vs. b2V2	0.1246	-1.298 to 1.547	0.9767	ns
V2b2 vs. V2R	-1.823	-3.235 to -0.4118	0.0073	**
b2V2 vs. V2R	-1.948	-3.38 to -0.5155	0.0044	**
stimulated conditions in ΔQ-GRK + GRK6				
V2b2 vs. b2V2	0.4999	-0.9585 to 1.958	0.6975	ns
V2b2 vs. V2R	-7.39	-8.801 to -5.978	<0.0001	****
b2V2 vs. V2R	-7.89	-9.358 to -6.421	<0.0001	****
