# Supplementary material for: Helix-bundle and C-terminal GPCR domains differentially influence GRK-specific functions and β-arrestin-mediated regulation
Source: Nat Commun. 2025 Jul 1;16:5430. doi: 10.1038/s41467-025-61281-4 (PMC12214593; doi:10.1038/s41467-025-61281-4)
Supplement: Supplementary file 2 — Description of Additional Supplementary Files [file 41467_2025_61281_MOESM2_ESM.pdf]

Description of Additional Supplementary Files

File name: Supplementary Movie 1
Description: Control cells were transfected with b2AR-CFP (blue), β-arrestin2-YFP (green) and Rab5-mCherry (red). Cells were stimulated at 0 sec (1 μM Iso) and imaged over 15 min after stimulation.

File name: Supplementary Movie 2
Description: Control cells were transfected with V2b2-CFP (blue), β-arrestin2-YFP (green) and Rab5-mCherry (red). Cells were stimulated at 0 sec (0.1 μM AVP) and imaged over 15 min after stimulation.

File name: Supplementary Movie 3
Description: Control cells were transfected with b2V2-CFP (blue), β-arrestin2-YFP (green) and Rab5-mCherry (red). Cells were stimulated at 0 sec (1 μM Iso) and imaged over 15 min after stimulation.

File name: Supplementary Movie 4
Description: Control cells were transfected with V2R-CFP (blue), β-arrestin2-YFP (green) and Rab5-mCherry (red). Cells were stimulated at 0 sec (0.1 μM) and imaged over 15 min after stimulation.
