# Supplementary material for: Helix-bundle and C-terminal GPCR domains differentially influence GRK-specific functions and β-arrestin-mediated regulation
Source: Nat Commun. 2025 Jul 1;16:5430. doi: 10.1038/s41467-025-61281-4 (PMC12214593; doi:10.1038/s41467-025-61281-4)
Supplement: Supplementary file 7 — Reporting Summary [file 41467_2025_61281_MOESM7_ESM.pdf]

nature portfolio

Corresponding author(s): Carsten Hoffmann

Last updated by author(s): May 22, 2025

Reporting Summary

Nature Portfolio wishes to improve the reproducibility of the work that we publish. This form provides structure for consistency and transparency in reporting. For further information on Nature Portfolio policies, see our Editorial Policies and the Editorial Policy Checklist.

Statistics

For all statistical analyses, confirm that the following items are present in the figure legend, table legend, main text, or Methods section.

n/a	Confirmed	
☐	☒	The exact sample size (*n*) for each experimental group/condition, given as a discrete number and unit of measurement
☐	☒	A statement on whether measurements were taken from distinct samples or whether the same sample was measured repeatedly
☐	☒	The statistical test(s) used AND whether they are one- or two-sided<br>*Only common tests should be described solely by name; describe more complex techniques in the Methods section.*
☒	☐	A description of all covariates tested
☐	☒	A description of any assumptions or corrections, such as tests of normality and adjustment for multiple comparisons
☐	☒	A full description of the statistical parameters including central tendency (e.g. means) or other basic estimates (e.g. regression coefficient) AND variation (e.g. standard deviation) or associated estimates of uncertainty (e.g. confidence intervals)
☐	☒	For null hypothesis testing, the test statistic (e.g. *F*, *t*, *r*) with confidence intervals, effect sizes, degrees of freedom and *P* value noted<br>*Give P values as exact values whenever suitable.*
☒	☐	For Bayesian analysis, information on the choice of priors and Markov chain Monte Carlo settings
☒	☐	For hierarchical and complex designs, identification of the appropriate level for tests and full reporting of outcomes
☒	☐	Estimates of effect sizes (e.g. Cohen's *d*, Pearson's *r*), indicating how they were calculated

Our web collection on statistics for biologists contains articles on many of the points above.

Software and code

Policy information about availability of computer code

Data collection	Plate reader data was obtained using a BioTek Synergy Neo2 reader with the Gen5 software version 2.09; confocal microscopy data was obtained using a Leika DMi8 TCS SP8 with the Leica Apllication SuiteX, version 3.5.5.19976; quantification of Western Blot images was done using Fujifilm Multi Gauge software (V3.0); the 7TM phosphorylation assay was performed using a Molecular Devices FlexStation 3 microplate reader using SoftMax Pro 5.4 software.
Data analysis	For data analysis, the following softwares were used: Microsoft office Excel 16.0, GraphPad Prism 7.03 (for the 7TM phosphorylation assay analysis version 9.3.1 was used), ImageJ Version 1.53p, the Squassh and SquasshAnalyst software (described in methods section and references by A. Rizk et al.), Python 3.11, R 4.2.2 including drc R package version 3.0-1 and pheatmap R package by Kolde et al. 2013. All code is made available here: https://github.com/mo-yoda/Matthees_fingerprints

For manuscripts utilizing custom algorithms or software that are central to the research but not yet described in published literature, software must be made available to editors and reviewers. We strongly encourage code deposition in a community repository (e.g. GitHub). See the Nature Portfolio guidelines for submitting code & software for further information.

Data

Policy information about availability of data

All manuscripts must include a data availability statement. This statement should provide the following information, where applicable:

- Accession codes, unique identifiers, or web links for publicly available datasets
- A description of any restrictions on data availability
- For clinical datasets or third party data, please ensure that the statement adheres to our policy

All data can be obtained from the corresponding author upon reasonable request.

Research involving human participants, their data, or biological material

Policy information about studies with human participants or human data. See also policy information about sex, gender (identity/presentation), and sexual orientation and race, ethnicity and racism.

Reporting on sex and gender	N/A
Reporting on race, ethnicity, or other socially relevant groupings	N/A
Population characteristics	N/A
Recruitment	N/A
Ethics oversight	N/A

Note that full information on the approval of the study protocol must also be provided in the manuscript.

Field-specific reporting

Please select the one below that is the best fit for your research. If you are not sure, read the appropriate sections before making your selection.

☒ Life sciences ☐ Behavioural & social sciences ☐ Ecological, evolutionary & environmental sciences

For a reference copy of the document with all sections, see nature.com/documents/nr-reporting-summary-flat.pdf

Life sciences study design

All studies must disclose on these points even when the disclosure is negative.

Sample size	All plate reader experiments were performed as three (or more where indicated) independent experiments with independent transfections and readings. Each datapoint was measured as three technical replicates. Western blots were quantified from membranes of freshly prepared lysates that were subjected to immunoprecipitations as indicated. Three sets of independent cell seedings and stimulations were prepared. The 7TM phosphorylation assay was performed as five independent experiments. Confocal microscopy was performed on three to four measurement days with independent transfections and multiple independent ligand stimulations per experimental day. A minimum of 28 individual images were analysed for each condition.
Data exclusions	Outliers were excluded. Fitted concentration-dependent conformational change data with an absolute value of the Hill slope smaller than 0.1 and EC50 values outside the range of 10^-3 to 10^0.3 µM were classified as non-responding and assigned the value of zero net BRET change.
Replication	All experiments could be reproduced as shown and repetitions are described above and in the respective methods section or figure legends.
Randomization	N/A
Blinding	N/A

Reporting for specific materials, systems and methods

We require information from authors about some types of materials, experimental systems and methods used in many studies. Here, indicate whether each material, system or method listed is relevant to your study. If you are not sure if a list item applies to your research, read the appropriate section before selecting a response.

Materials & experimental systems

n/a	Involved in the study	
☐	☒	Antibodies
☐	☒	Eukaryotic cell lines
☒	☐	Palaeontology and archaeology
☒	☐	Animals and other organisms
☒	☐	Clinical data
☒	☐	Dual use research of concern
☒	☐	Plants

Methods

n/a	Involved in the study	
☒	☐	ChIP-seq
☒	☐	Flow cytometry
☒	☐	MRI-based neuroimaging

Antibodies

Antibodies used	anti HA-antibody (Biolegend, #682404),<br>anti-vinculin (BIOZOL, BZL03106),<br>anti-pERK phospho-p44/42 (Cell signaling technology, #9106),<br>total ERK: p44/42 (Cell signaling technology, #9107),<br>goat anti-rabbit (SeraCare, #5220-0336),<br>goat anti-mouse (SeraCare, #5220-0341);<br>for the 7TM phosphorylation assay:<br>b2AR anti-pS355/pS356-β2 (7TM Antobodies, 7TM0029A),<br>b2AR anti-pT360/pS364-β2 (7TM Antibodies, 7TM0029B),<br>V2R anti-pT359/pT360-V2 (7TM Antibodies, 7TM0368B),<br>V2R anti-pS362/pS363/pS364-V2 (7TM Antibodies, 7TM0368C),<br>rabbit polyclonal anti-HA antibody (7TM Antibodies, 7TM000HA),<br>Anti-rabbit HRP-linked antibody (Cell Signaling Technology, #7074)
Validation	https://www.biolegend.com/de-de/products/alexa-fluor-647-anti-ha-11-epitope-tag-antibody-12506?GroupID=GROUP26,<br>Reichel et al. 2022 (doi: 10.3390/ijms23031195),<br>https://www.cellsignal.com/products/primary-antibodies/phospho-p44-42-mapk-erk1-2-thr202-tyr204-e10-mouse-mab/9106?srsltid=AfmBOorzHBnD8TrsRPKN3rr5S4nj0M-CkhhvYZbxrKbkm_CUzylsO-Vu,<br>https://www.cellsignal.com/products/primary-antibodies/p44-42-mapk-erk1-2-3a7-mouse-mab/9107,<br>https://www.seracare.com/AntiRabbit-IgG-HL-Antibody-PeroxidaseLabeled-5220-0336/,<br>https://www.seracare.com/AntiMouse-IgG-HL-Antibody-Human-Serum-Adsorbed-and-PeroxidaseLabeled-5220-0341/,<br>https://7tmantibodies.com/phosphosite-7tm-antibodies/adrenoceptors/v2-adrenoceptor/107/ps355/ps356-v2-phospho-v2-adrenoceptor-antibody,<br>https://7tmantibodies.com/phosphosite-7tm-antibodies/adrenoceptors/v2-adrenoceptor/108/pt360/ps364-v2-phospho-v2-adrenoceptor-antibody,<br>https://7tmantibodies.com/phosphosite-7tm-antibodies/vasopressinoxytocin-receptors/v2-receptor/358/pt359/pt360-v2-phospho-vasopressin-receptor-2-antibody,<br>https://7tmantibodies.com/phosphosite-7tm-antibodies/vasopressinoxytocin-receptors/v2-receptor/359/ps362/ps363/ps364-v2-phospho-vasopressin-receptor-2-antibody,<br>https://7tmantibodies.com/ihc-grade-7tm-antibodies/epitope-tag-antibodies/ha/21/ha-tag-anti-ha-epitope-tag-antibody,<br>https://www.cellsignal.com/products/secondary-antibodies/anti-rabbit-igg-hrp-linked-antibody/7074?srsltid=AfmBOoqQtVGYO-eOoBX3O6JjIdiubzB7HXqg65vIBWlz-6odtJjubye8

Eukaryotic cell lines

Policy information about cell lines and Sex and Gender in Research

Cell line source(s)	HEK293 cells were originally obtained from DSMZ Germany (ACC 305). The GRK knockout cells were generated in house as described in Drube et al. 2022 (doi: 10.1038/s41467-022-28152-8). The stable cell lines were generated as described in the manuscript.
Authentication	The authors did not further authenticate the cells.
Mycoplasma contamination	All used cell lines were checked for mycoplasma infection regularly using the LONZA MycoAlert mycoplasma detection kit (LT07-318) and were found to be negative.
Commonly misidentified lines<br>(See ICLAC register)	N/A

Plants

Seed stocks	*Report on the source of all seed stocks or other plant material used. If applicable, state the seed stock centre and catalogue number. If plant specimens were collected from the field, describe the collection location, date and sampling procedures.*
Novel plant genotypes	*Describe the methods by which all novel plant genotypes were produced. This includes those generated by transgenic approaches, gene editing, chemical/radiation-based mutagenesis and hybridization. For transgenic lines, describe the transformation method, the number of independent lines analyzed and the generation upon which experiments were performed. For gene-edited lines, describe the editor used, the endogenous sequence targeted for editing, the targeting guide RNA sequence (if applicable) and how the editor was applied.*
Authentication	*Describe any authentication procedures for each seed stock used or novel genotype generated. Describe any experiments used to assess the effect of a mutation and, where applicable, how potential secondary effects (e.g. second site T-DNA insertions, mosiacism, off-target gene editing) were examined.*
