# Supplementary figures and images for: Helix-bundle and C-terminal GPCR domains differentially influence GRK-specific functions and β-arrestin-mediated regulation
Source: Nat Commun. 2025 Jul 1;16:5430. doi: 10.1038/s41467-025-61281-4 (PMC12214593; doi:10.1038/s41467-025-61281-4)

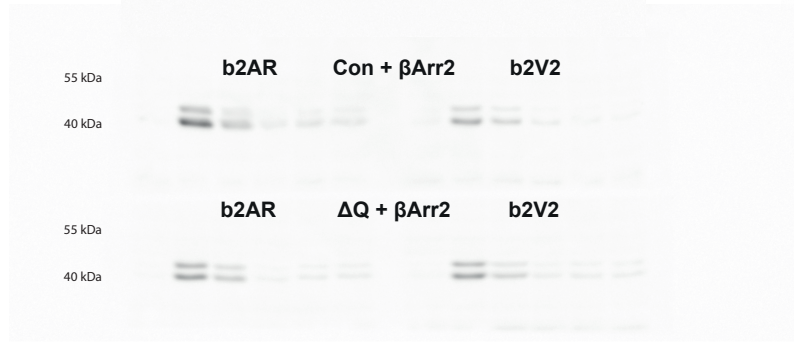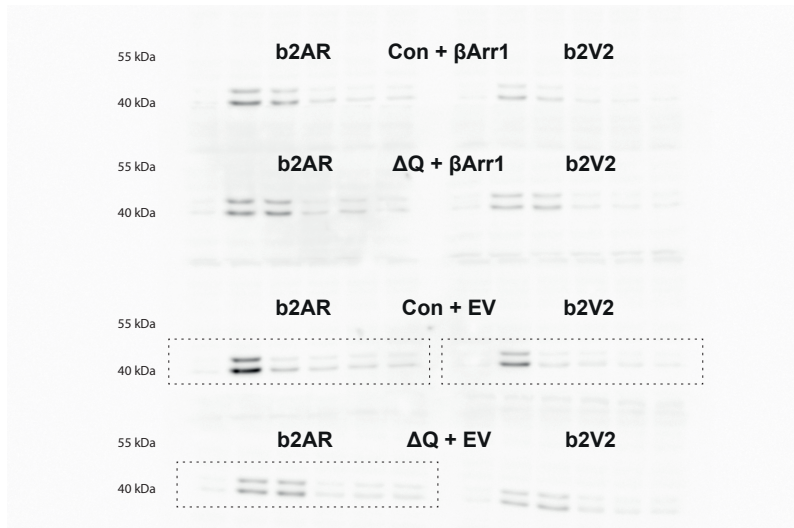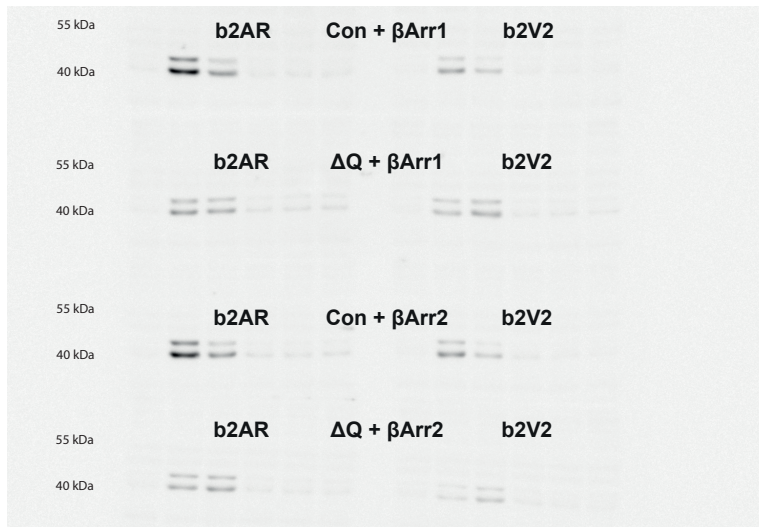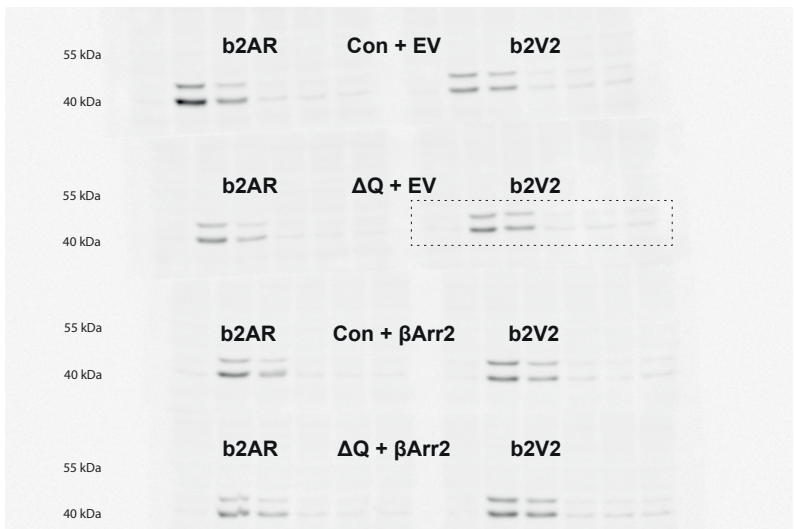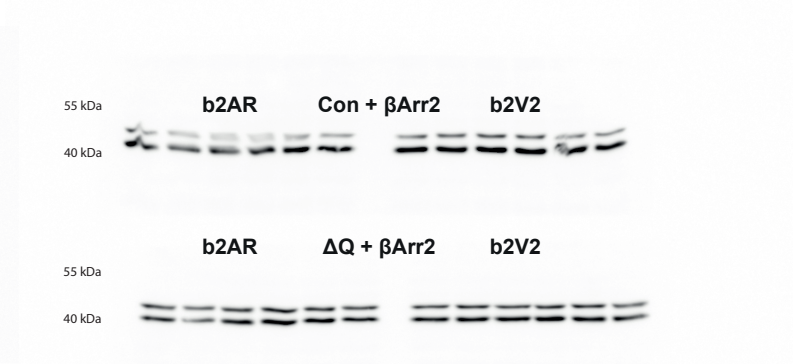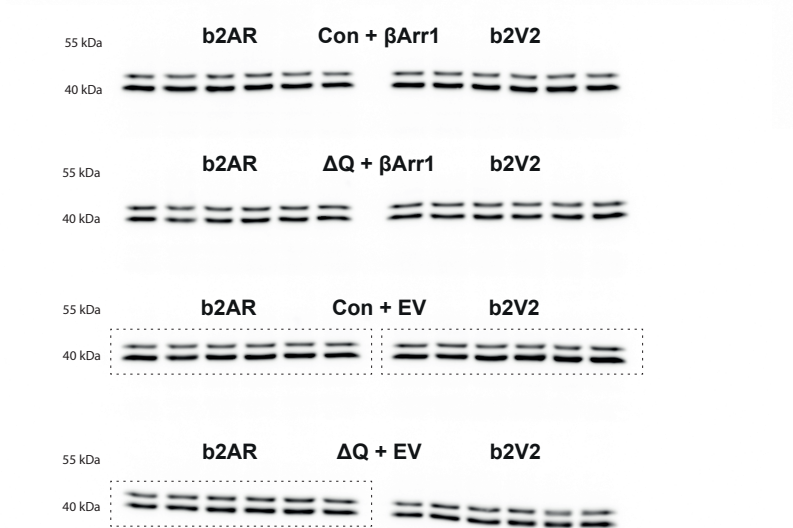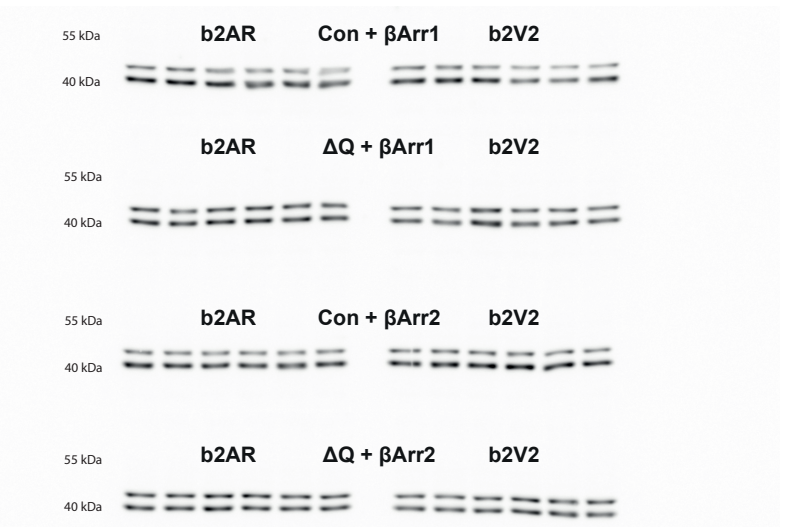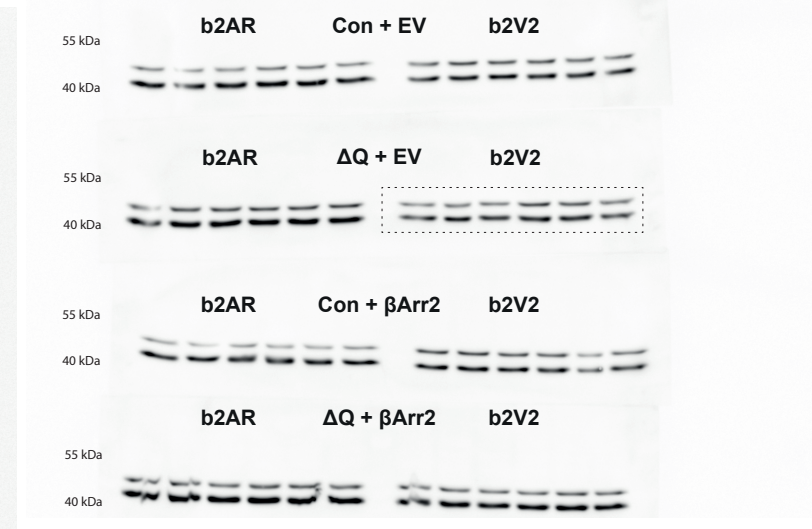

Supplement: Supplementary file 9 — Source Data [file 41467_2025_61281_MOESM9_ESM.zip › ERK blots all replicates b2AR,b2V2.pdf]

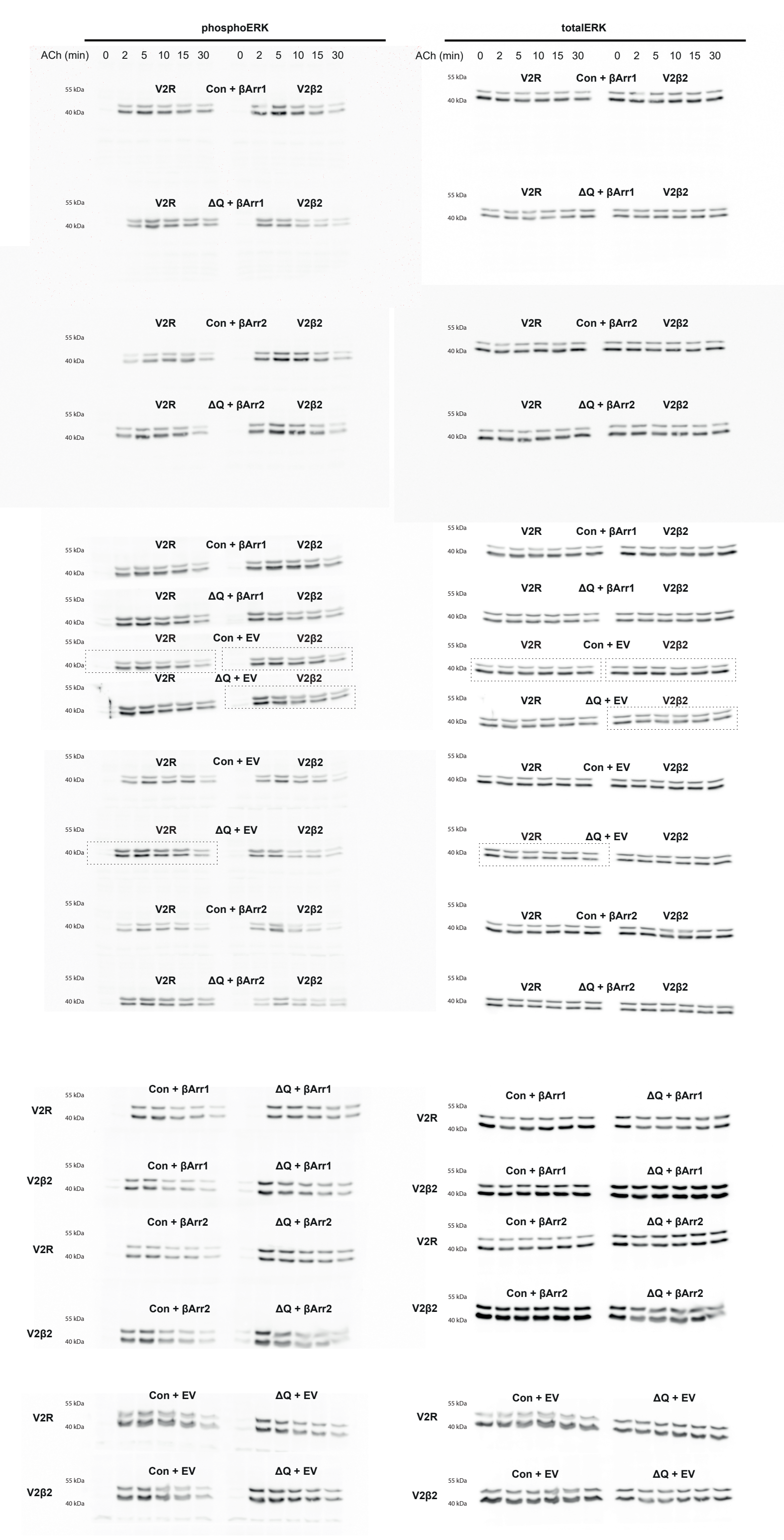

Supplement: Supplementary file 9 — Source Data [file 41467_2025_61281_MOESM9_ESM.zip › ERK blots all replicates V2R, V2b2.pdf]
